# Mineralogy and Geochemistry of the Main Glauconite Bed in the Middle Eocene of Texas: Paleoenvironmental Implications for the Verdine Facies

**DOI:** 10.1371/journal.pone.0087656

**Published:** 2014-02-04

**Authors:** Sherie C. Harding, Barbara P. Nash, Erich U. Petersen, A. A. Ekdale, Christopher D. Bradbury, M. Darby Dyar

**Affiliations:** 1 Department of Geology and Geophysics, University of Utah, Salt Lake City, Utah, United States of America; 2 Department of Astronomy, Mount Holyoke College, South Hadley, Massachusetts, United States of America; The Pennsylvania State University, United States of America

## Abstract

The Main Glauconite Bed (MGB) is a pelleted greensand located at Stone City Bluff on the south bank of the Brazos River in Burleson County, Texas. It was deposited during the Middle Eocene regional transgression on the Texas Gulf Coastal Plain. Stratigraphically it lies in the upper Stone City Member, Crockett Formation, Claiborne Group. Its mineralogy and geochemistry were examined in detail, and verdine facies minerals, predominantly odinite, were identified. Few glauconitic minerals were found in the green pelleted sediments of the MGB. Without detailed mineralogical work, glaucony facies minerals and verdine facies minerals are easily mistaken for one another. Their distinction has value in assessing paleoenvironments. In this study, several analytical techniques were employed to assess the mineralogy. X-ray diffraction of oriented and un-oriented clay samples indicated a clay mixture dominated by 7 and 14Å diffraction peaks. Unit cell calculations from XRD data for MGB pellets match the odinite-1M data base. Electron microprobe analyses (EMPA) from the average of 31 data points from clay pellets accompanied with Mössbauer analyses were used to calculate the structural formula which is that of odinite: Fe^3+^_0.89_ Mg_0.45_ Al_0.67_ Fe^2+^_0.30_ Ti_0.01_ Mn_0.01_)_ Σ = 2.33_ (Si_1.77_ Al_0.23_) O_5.00_ (OH)_4.00_. QEMSCAN (Quantitative Evaluation of Minerals by Scanning Electron Microscopy) data provided mineral maps of quantitative proportions of the constituent clays. The verdine facies is a clay mineral facies associated with shallow marine shelf and lagoonal environments at tropical latitudes with iron influx from nearby runoff. Its depositional environment is well documented in modern nearshore locations. Recognition of verdine facies clays as the dominant constituent of the MGB clay pellets, rather than glaucony facies clays, allows for a more precise assessment of paleoenvironmental conditions.

## Introduction

Green clay-rich sediment is recognized throughout the geologic record, and the paleoenvironmental implications of green clay facies are not always understood clearly. This study reports the clay mineralogy and geochemistry of the Main Glauconite Bed (MGB), a shelly and sandy mudstone that has an olive-gray hue and is bioturbated, pelleted, and trace fossiliferous, with diverse body fossils. These characteristics, especially the abundant ovoidal, small clay pellets, probably led to its being named the Main Glauconite Bed. However, very few glauconitic minerals are present in the MGB.

**Figure 1:**
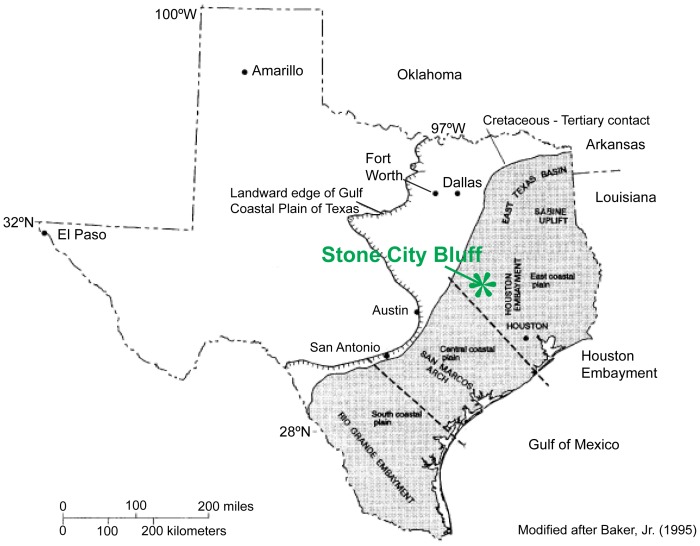
Location of the Stone City Bluff study area on the Texas Gulf Coastal Plain [59].

**Figure 2:**
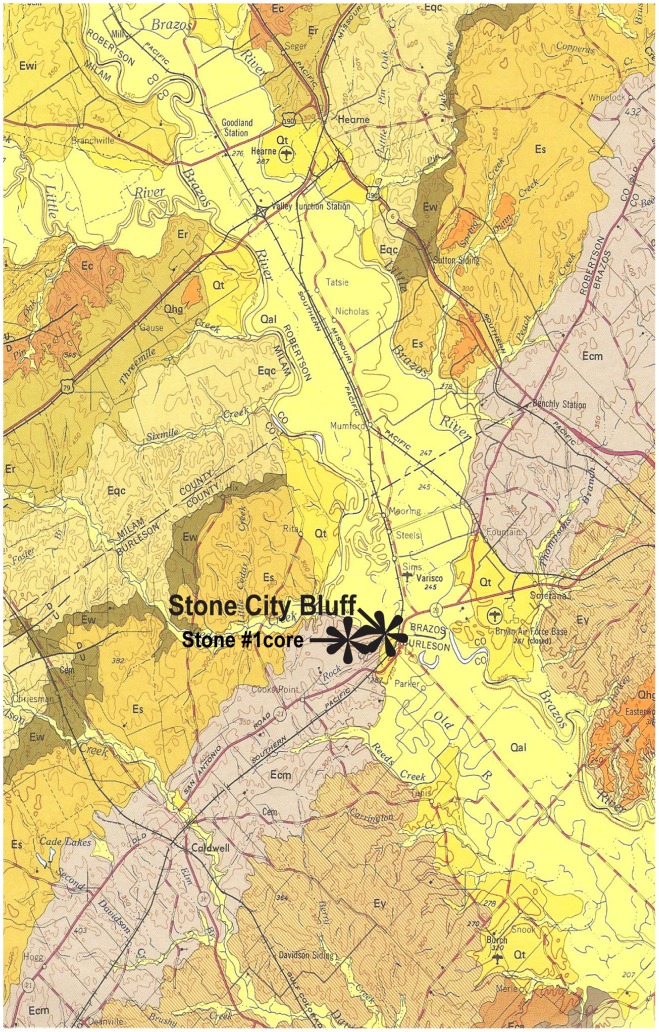
Geologic Atlas of Texas, Austin Sheet, Burleson and Brazos Counties [60]. Stone City Bluff and Stone #1core located. Wilcox Group (Ewi); Crockett Fm., also known as the Cook Mountain Fm. (Ecm); and Caddell Fm. of the Jackson Group (Eca).

**Figure 3:**
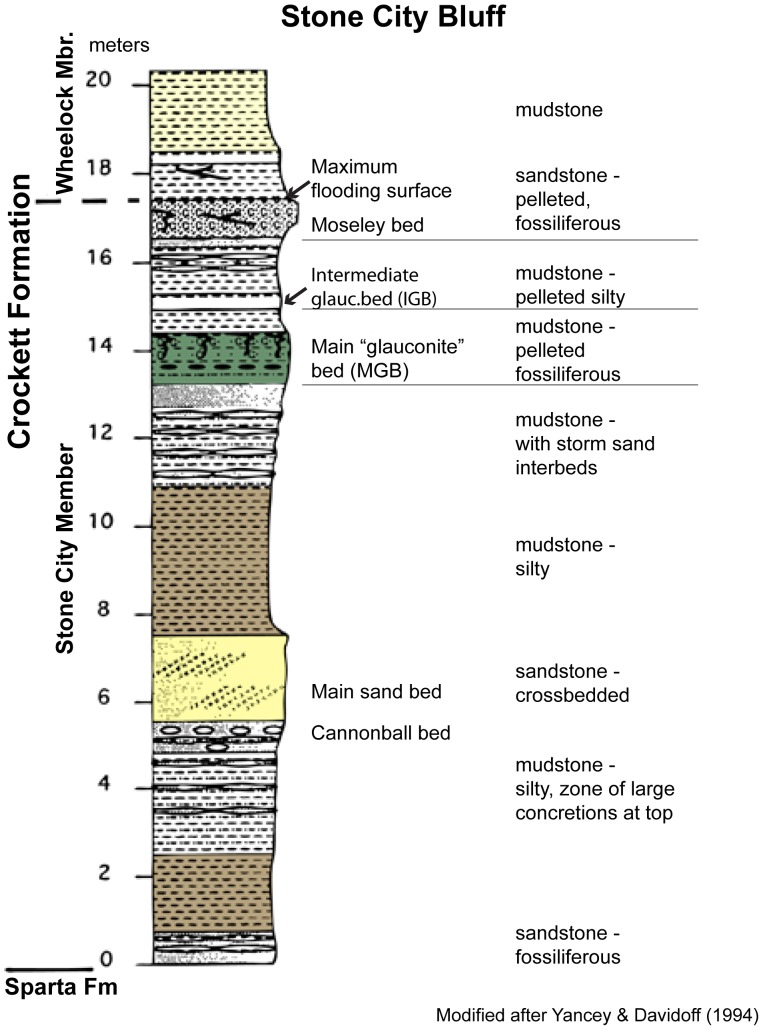
Middle Eocene stratigraphic section includes Stone City Member, Crockett Formation, Claiborne Group (modified after Yancey [6]).

**Figure 4:**
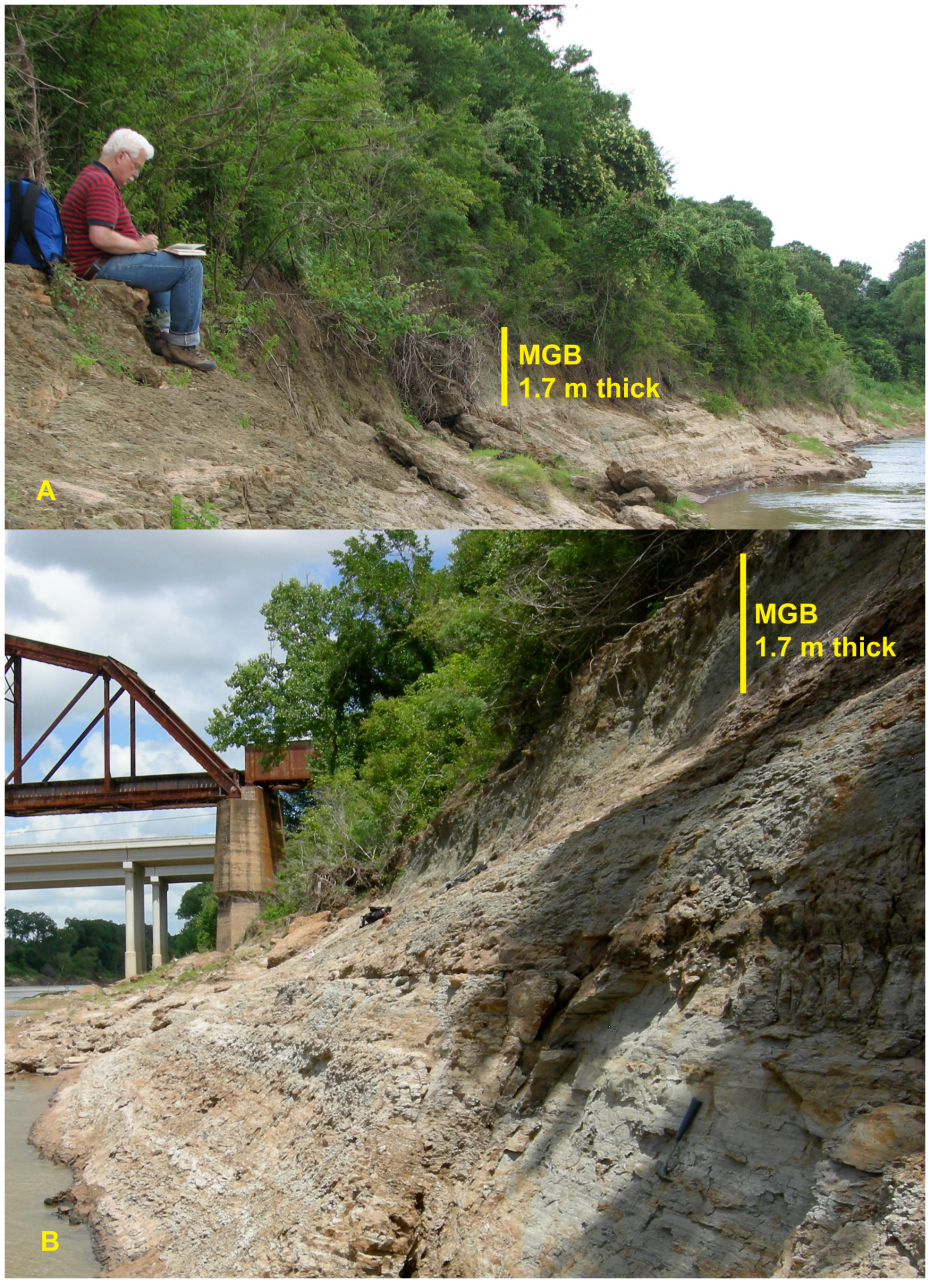
Stone City Bluff outcrop photos, the MGB is green and nearly vertical, A) looking west, upstream (the subject in this photograph has given written informed consent, as outlined in the PLOS consent form, to publication of their photograph), B) looking east, downstream.

**Figure 5:**
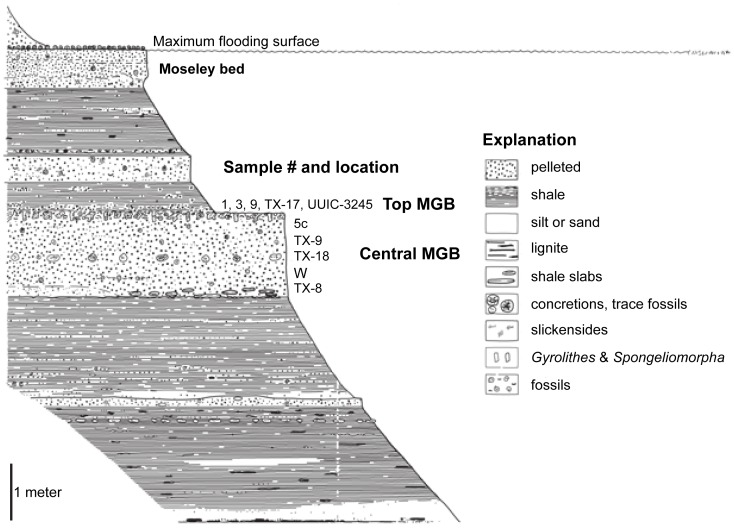
Upper units of the Stone City Member, sample number and location (modified after Stenzel [2]).

The study area, Stone City Bluff, is located on the Gulf of Mexico Coastal Plain in east-central Texas (Figure 1). A detailed geologic map (Figure 2) shows the underlying Wilcox Group, formations of the Claiborne Group, and overlying formations of the Jackson Group. The Crockett Formation is also known as Cook Mountain Formation. The Middle Eocene, stratigraphic section (Figure 3) includes the Stone City Member, Crockett Formation. The upper part of the section outcrops along the south bank of the Brazos River in Burleson County, where the MGB is a prominent 1.7 m thick greensand unit. The MGB in outcrop at Stone City Bluff is pictured in Figure 4. Sample locations in the central zone and concretionary burrows at the top of the MGB are shown (Figure 5).

The mineralogy and geochemistry of the central zone and top layer of the MGB are different. The central part of the MGB contains heterogeneous green marine clay that is intensely bioturbated with a significant fraction of fecal pellets. The top part of the MGB is lined with concretions of filled burrows that extend down into the central MGB. These concretions are composed of siderite and apatite that formed as cementing agents. The green minerals in the central zone of the MGB warrant close examination, as do the contrasting minerals at the top of the MGB, because they reflect a change in the depositional environment.

### Previous Work on the MGB

The MGB and associated stratigraphy have been studied extensively by other workers who have offered various mineralogical and paleoenvironmental interpretations [1]–[9]. Thornton [10] reported, in a master’s thesis, that the green marine clay of the MGB had the characteristics of odinite. Huggett et al. [11] described the geochemistry of authigenic minerals in the Claiborne Group, including samples from Stone City Bluff, and concluded that the composition was dark green serpentine-rich mixed layer clay. This characterization of the clay mineralogy and geochemistry of the MGB clay pellets, as well as its ichnology (evidence from trace fossils), have not received detailed treatment until now. It contributes to a coordinated approach to understanding the complex processes during accumulation of the MGB.

**Figure 6:**
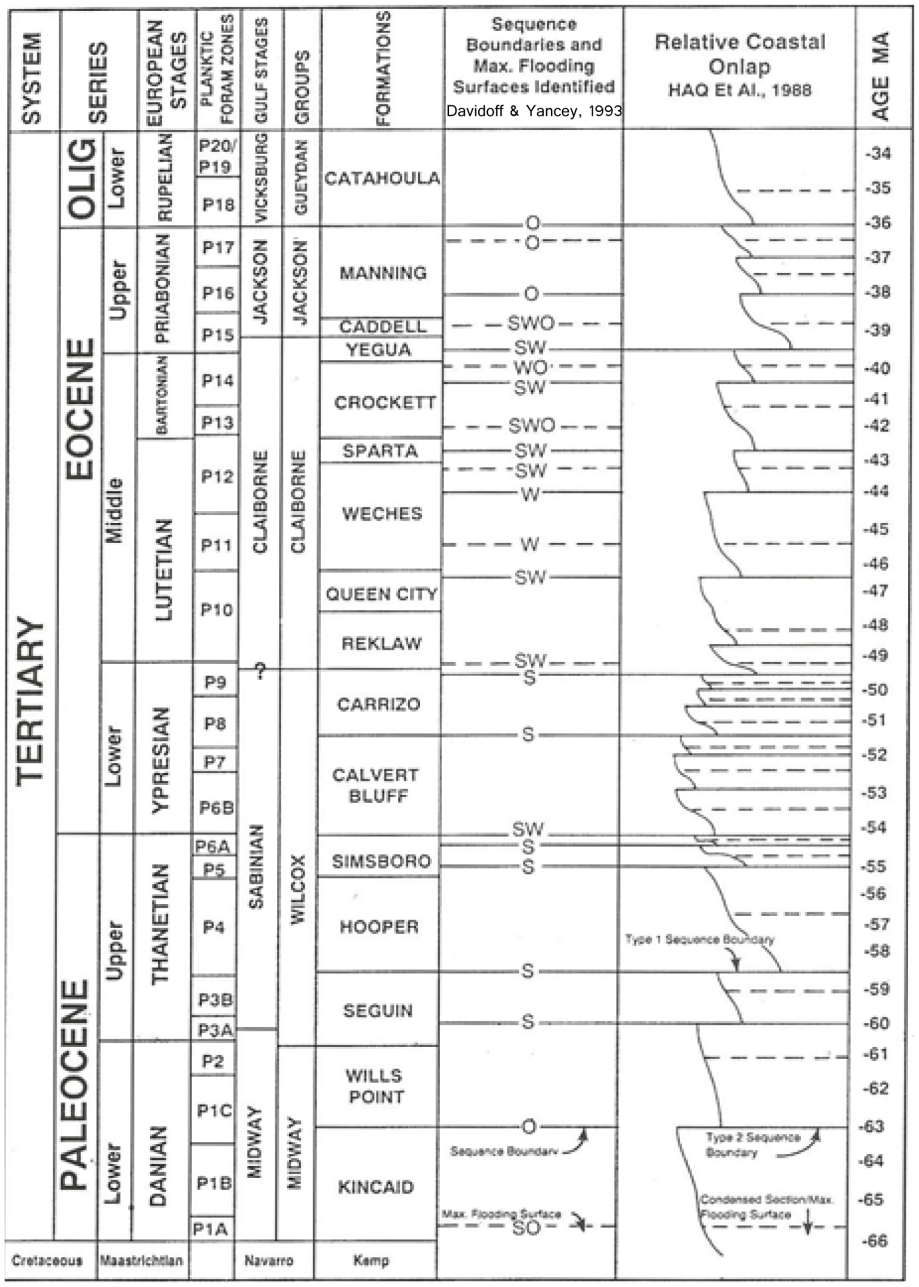
Stratigraphic chart and related lithostratigraphy of the Brazos River Valley, major sequence boundaries (solid lines) and maximum flooding surfaces (dashed lines), identified by S = seismic; W = well logs; O = outcrop (modified after Davidoff & Yancey [7]).

Previous interpretations of the depositional environment of the Stone City Member suggest that it was dynamic and complex. Davidoff and Yancey [12] documented eustatic sea-level changes and identified major sequence boundaries and maximum flooding surfaces in the Middle Eocene and some parasequences that may be capped by an exposure surface. The regional sequence stratigraphy testifies to numerous sea-level fluctuations during the early Tertiary (Figure 6). Stanton and Nelson [13] and Zuschin and Stanton [9] reconstructed the paleocommunity of the MGB, and they identified a time-averaged, parautochthonous, highly diverse fossil assemblage in the central bed along with a complex sedimentologic and taphonomic history due to reworking by winnowing and bioturbation. Stanton and Warme [3] examined the trace fossils at Stone City Bluff, and they suggested an interfingering of off-delta transgression of the sea into interdeltaic bays and sounds, marked by a gradual change from restricted and perhaps brackish delta-margin environments and biota to a normal marine environment and biota. Berg [14] interpreted the Stone City section as restricted marine or lagoonal environment. Yancey [6] reported on depositional trends of the Stone City transgressive-systems-tract, and he suggested that it is a transgressive depositional system in an area characterized by high sedimentation rate during progressive marine deepening. He proposed that the Stone City Bluff section is capped by a maximum flooding surface of regional extent. Previous interpretations of paleoenvironment are varied. The mineralogic and geochemical findings in this study offer further information and complement findings in previous studies.

### Background on Green Clay Minerals

#### Green clay minerals

Published research on green minerals in sedimentary rocks reveals a lack of consistency in use of mineralogic terms. Terms such as “glauconite,” “greensands,” “glaucony,” or “glauconitic minerals,” are used frequently in the literature, and confusion persists. Glauconite, strictly speaking, is a series name for dioctahedral, interlayer-deficient micas with compositions of K_0.8_R^3+^_1.33_R^2+^_0.67_Al_0.13_(Si_3.87_)O_10_(OH)_2_ [15]. The term also has been employed as a common field term for small, rounded, green to black pellets and grains. Field identification often was confined to morphological appearance and color. In this report, as suggested by Odin [16], the term “glauconitic minerals” is used for the minerals that are rich in Fe^3+^ with a potassium content greater than 2 wt.% K_2_O in the interlayer. In the case of the MGB, the few glauconitic minerals detected were usually confined to sparse, isolated grains.

Numerous studies have attempted to characterize clay mineralogy in marine sedimentary environments [11], [16]–[24]. Odin’s treatise on *Green Marine Clays* inventories green minerals in the modern marine environment and separates green marine clays into four facies: oolitic ironstone facies, verdine facies, glaucony facies and celadonite-bearing facies, based on examples on today’s ocean floors. Odin [16] demonstrated that clay minerals on the sea floor progressively transform by slow cation exchange. The term “glaucony” is introduced to designate the fully marine facies characterized by a green pigment made of 2∶1 type clay minerals that include glauconitic minerals, which form today at water depths around 50 m to as much as 1000 m on the sea floor.

The verdine facies is represented by a variety of chlorite-like clays, with an alternative mineralogy and geological significance. The verdine facies is identified in modern, shallow, tropical environments, in proximity to a terrigenous clastic source with iron influx. It is characteristic of bottom sediments of nearly normal salinity, but at a shallower depth range of 15 to 60 m. Mineralogically, verdine facies clays are uniquely different from those in the glaucony facies. Glauconitic minerals are absent from the verdine facies, while 7 and 14Å clays are present. Odin identified new clay minerals for the verdine facies, initially termed phyllite-V and phyllite-C clays, and later named odinite and iron smectite [16], [24].

#### Authigenesis

Mineral authigenesis in sea-floor sediments can occur as glauconitization or verdinization, processes by which a sea-floor substrate is progressively modified to glauconitic minerals or to verdine minerals, respectively [16]. These result from interaction between open seawater and sea-floor sediment, and they are influenced by latitude, sea-floor temperature and bathymetric setting on the continental shelf.

Glauconitization results in a continuum of minerals from K-poor, disordered glauconitic smectite to K-rich, ordered glauconitic mica. Stages of development are defined by increasing potassium oxide content where 2 wt.%–4 wt.% K_2_O is nascent stage, 4 wt.% -6 wt.% K_2_O is slightly evolved (slightly mature), 6 wt.%–8 wt.% K_2_O is evolved (mature), and greater than 8 wt.% K_2_O is highly evolved (highly mature) glauconitic minerals [16], [21], [25]–[29]. The level of maturity is a reflection of residence time on the sea floor in sediment-starved conditions. Potassium, at 0.4 parts per thousand (ppt), is one of six major dissolved constituents in normal seawater [30]. It is available for uptake during glauconitization. Evolution to the highly mature stage takes 10^5^ to 10^6^ years in recent material [16]. Optimal conditions for glauconitization include a sea-floor temperature of 10–15°C, and unrestricted seawater circulation, allowing for winnowing accompanied by a slow sediment accumulation rate. Glauconitization commonly occurs in a substrate rich in fecal pellets and/or foraminiferal tests, which provide micro-reducing environments favorable for glauconitization. Oxidizing conditions may prevail in the surrounding substrate due to winnowing currents. Glauconitic minerals commonly are associated with body fossils and trace fossils indicating sufficient oxygen to support benthic life. The glauconitization process may be halted at any stage of the maturity continuum if the environment becomes unsuitable because of a change in sea level or burial depth. A high rate of detrital influx will inhibit or entirely prevent glauconitization. Once formed, glauconitic minerals are highly stable and resistant to dissolution in the marine environment, which suggests that they can be reworked or transported with little degradation in a changing environment [16], [21], [25]–[29].

**Figure 7:**
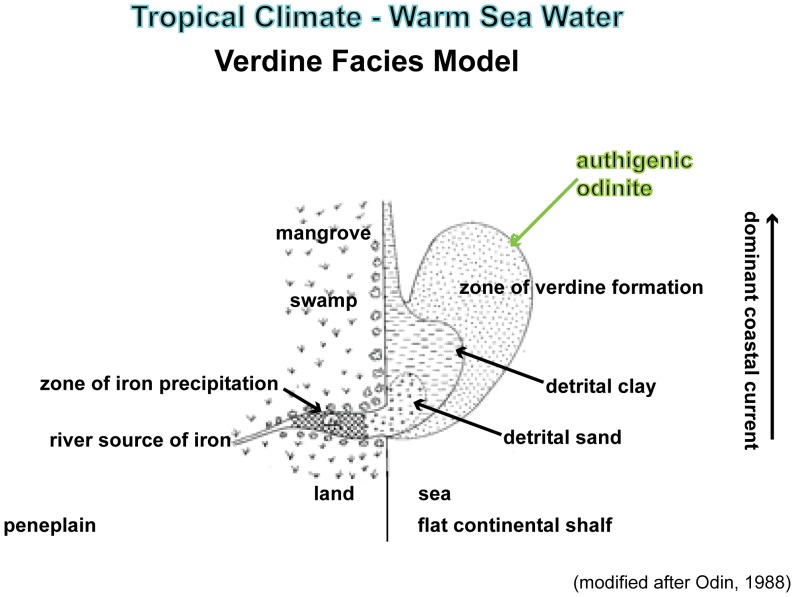
Diagrammatic verdine facies model depicting the idealized paleoenvironment at a tropical river mouth (modified after Odin [16]).

Verdinization, on the other hand, requires a shorter residence time on the sea floor [16]. Verdine facies minerals form rather quickly, probably in thousands of years, because of increased sea-floor temperature (∼25°C). They tend to occur in shallower water under normal salinity and basic pH (7.5–8.5). Observed water depth where verdine minerals are forming today is between 15 and 60 m, and locally in 5 m depths, in common association with fecal pellets. Circulating currents are required in proximity to continental water input and abundant Si, Mg, and Fe in tropical to subtropical, warm water environments (Figure 7) [16], [24], [32]. Verdinization progresses from light green to dark green clay minerals. Verdine facies clay minerals are comprised primarily of two clay mineral species characterized by 7.2 and 14.5Å x-ray diffraction peaks [16].

The predominant verdine mineral is odinite, which is an iron and magnesium 1∶1, 7Å clay. It is classified by some authors in the kaolin-serpentine group and the dioctahedral kaolin sub-group of phyllosilicates [15] or by other authors in the serpentine group [33]. Odinite was first described by Bailey [24] as serpentine-like, intermediate between dioctahedral and trioctahedral. It has total iron of about 75% Fe^3+^ and 25% Fe^2+^, and a nominal structural formula of (Fe^3+^,Mg,Al,Fe^2+^,Ti,Mn)_5_(Si,Al)_4_O_10_(OH)_8_ [15]. Odinite is commonly identified in modern environments and is considered a possible analog to the oolitic ironstone facies in ancient rocks [16], [24], [32]. Although few possible occurrences of odinite from the geologic record have been reported in the literature odinite, or its alteration products, may be more common than is generally recognized.

Verdinization and glauconitization are processes that may be influenced by biogenic activity. Sediment-starved shelf environments often are characterized by high biological productivity. Biogenic activity, such as recycling of seafloor sediments by deposit-feeding and suspension-feeding organisms, can be prevalent in green marine clay environments at shelf depths. The effect of biological activity on clay mineral authigenesis, mineral growth and/or silicate weathering, is significant [23]. The MGB is a highly bioturbated, pelleted and fossiliferous unit. During deposition, biogenic activity on the seafloor was intense [2]. The existing mineralogy and geochemistry of the MGB may have been affected by animal-sediment interaction.

#### Diagenesis and weathering

Diagenesis refers to the physical and chemical reactions occurring in sediment after burial. Glauconitic minerals may be modified after burial, often showing an Al increase concomitant with Fe decrease. During weathering, glauconitic minerals may be altered in color and chemistry. In one study, Pestitschek et al. [34] described degradation over time between fresh and weathered glauconite. They compared chemical results of Upper Cretaceous glauconitic minerals on the surface with core samples in the subsurface and demonstrated a loss of both Fe and K during weathering. They suggested that weathering reverses the glauconitization process, as glauconitic minerals degrade to smectite. They found that fresh samples were dark green, whereas the weathered samples were olive green to yellowish brownish green [34].

The MGB exhibits a variety of pellet colors, which may vary within a given pellet. This may be a function of different stages of diagenesis. Also, the possibility of weathering is suspected since the MGB is subaerially exposed. When mineralogical results of surface and core samples from the MGB were compared, there was no evidence of degradation due to weathering. The MGB showed similar mineral composition in both core and in outcrop samples. Therefore, it may be reasonably assumed that weathering is not an important influence on the MGB pellets, and that the lack of glauconitic minerals in the MGB is not due to weathering. Glauconitic minerals are not known to alter to odinite [16].

The fate of odinite, a verdine facies mineral, during diagenesis and weathering is not well known. According to Ku and Walter [32], odinite forms very quickly and apparently is metastable, because most of the reported occurrences of odinite are less than 20,000 years old. When odinite is exposed to fully oxygenated seawater or atmospheric oxygen, it changes color from grayish green to yellowish red, and it has been known to oxidize to goethite. Several workers have suggested the verdine facies as a modern precursor for ancient oolitic ironstones and grain-coating chlorite [16], [32]. Huggett et al. [11] suggest that serpentine-rich mixed-layer clays of the Claiborne Group may be diagenetically altered odinite, and that serpentine is intermediate between odinite and berthierine. Ryan and Hillier [35] suggest that odinite, or evolved verdine minerals, may be precursor to authigenic chlorite in the Jurassic Sundance Formation of Wyoming. Whether the MGB is comprised of authigenic verdine facies minerals or altered authigenic verdine minerals, the paleoenvironmental implications are essentially the same.

## Methods

### Sampling

The Stone City Bluff outcrop of the Crockett Formation is readily accessible for sampling. Concretionary burrows at top of the MGB can be removed easily due to the friable nature of the sediment. The upper section of Stone City Bluff, particularly the MGB, was sampled for this study. Twenty-seven samples were collected at 60 cm intervals from two measured sections. Concretions, representative of filled burrows, were collected from the top of the MGB and from tumbled blocks. Eight samples were retrieved from a drill core (Stone #1 core at Texas A & M University). These represent unweathered samples of the Stone City Member. Additional samples, including concretionary burrows and pelleted sediment, were made available by JE Warme (Colorado School of Mines) [3]. Polished thin sections were prepared, and standard analytical procedures were used as described below.

The sections were measured and samples were collected via the public access site at the highway bridge over the Brazos River. No specific permissions were required. The field studies did not involve any endangered or protected species. The project did not involve any non-human primates or any other vertebrae animals.

### Mineral Identification

Petrography of each thin section was described using a polarizing light microscope, and a variety of analytical techniques were employed to ascertain mineral composition and texture. X-ray diffraction (XRD) was carried out on oriented and unoriented samples to determine clay mineralogy. Electron microprobe (EMPA) analyses on selected pellets and matrix determined chemical composition and assisted with clay mineral identification. Mössbauer analyses were done on clay matrices and pellets to constrain mineralogy and determine iron oxidation state, which has bearing on EMPA data interpretation and significance for paleoenvironmental interpretation. QEMSCAN (Quantitative Evaluation of Minerals by Scanning Electron Microscopy) data were obtained to provide mineral maps and a textural view that complements, enhances, validates and quantifies the petrographic observations.

#### X-ray diffraction analysis

Clay minerals were examined from five central MGB samples (TX-8, W, TX-18, TX-9 & 5c) by X-ray diffraction (XRD) analyses. The 2 µm clay fraction was extracted by crushing, dispersion, and two-stage centrifuge. Oriented clay mounts were prepared and analyzed under conditions of air dried, ethylene glycolated, and heated to 375°C, then to 500°C for one hour. These were analyzed at 2° per minute from zero to 30° 2 θ, and examined in composite diffractograms. Randomly oriented powders were prepared by gently hand crushing bulk samples from TX-8 & TX-9 comprised of pellets and clay matrix from the central MGB. Pellets in samples 5c and TX-MGB were concentrated using a Frantz Magnetic Separator. TX-MGB is comprised of pellets from TX-8, W & TX-9. These were gently hand crushed and side loaded into an aluminum holder for analysis of unoriented powder. They were then analyzed at 2° per minute from zero to 66°. Eleven (11) peaks were indexed in the refinement, and quartz served as an internal standard. Selected samples were examined at high resolution, 0.5° per minute from 56° to 66° 2θ.

#### Electron microprobe analysis

Thirty clay pellets from both the central MGB and the concretionary burrow fill at the top of the MGB, and 14 non-pellet, cement areas were analyzed with a Cameca SX-50 electron microprobe equipped with four wavelength-dispersive spectrometers. Clay pellets were selected for analysis based on color in thin section and false color in QEMSCAN images. In an attempt to locate a potassium X-ray signal, some spot analyses focused on QEMSCAN-identified glauconitic grains, although very sparse. The more numerous clay pellets showed a low potassium signal. One to four spots were analyzed on each pellet for a total of 44 clay analyses. Analytical conditions were 15 keV accelerating voltage, 20 nA beam current, and a defocussed beam of 10–20 µm in diameter. A suite of natural minerals was employed as standards, and X-ray intensities were reduced using a phi-rho-z algorithm [36].

#### Mössbauer spectroscopy

Mössbauer analyses were used to constrain mineralogy and establish the iron oxidation state of pellets and matrix. The slightly magnetic pellets were separated from matrix in three central MGB samples (W, TX-9 & 5c) using a Frantz Magnetic Separator. Concentrated pellets and pellet free matrix were analyzed.

Sample mounts were prepared by gently mixing 30–40 mg of powdered sample with sugar to reduce preferred orientation. The mixtures were placed in a sample holder confined by Kapton tape. Mössbauer spectra were acquired at 295K using a source of ∼40 mCi ^57^Co in Rh on a WEB Research Co. model WT302 spectrometer. For each sample, the fraction of the baseline due to the Compton scattering of 122 keV gammas by electrons inside the detector was determined by measuring the count rate with and without a 14.4-keV stop filter (∼2 mm of Al foil) in the gamma beam. Compton-corrected absorption was calculated for each individual spectrum using the formulation *A*/(1–*b*), where *b* is the Compton fraction and *A* is the uncorrected absorption. Run times were 6–48 hours for each spectrum, and baseline counts were ∼6–14 million after the Compton correction, as needed to obtain reasonable counting. Data were collected in 1024 channels and corrected for nonlinearity via interpolation to a linear velocity scale, which is defined by the spectrum of the 25 µm Fe foil used for calibration. Data then were folded before fitting.

Data were modeled using an in-house program from the University of Ghent, Belgium, called DIST_3E (an implementation of software described in Wivel and Mørup [37]), which uses model-independent quadrupole splitting distributions for which the subspectra are constituted by Lorentzian shaped lines. This program does not presume any particular shape of the distribution, in contrast to other distribution programs (e.g., Recoil). All Mössbauer data are posted for public use at http://www.mtholyoke.edu/courses/mdyar/database/.

In all fits, isomer shift (IS) and quadrupole splitting (QS) of the doublets were allowed to vary, and widths of both peaks in each pair were coupled to vary in unison (i.e. one width for each doublet, but every doublet independent). In a few cases it was necessary to constrain peak widths to lie above a certain value to obtain reasonable parameters, but most spectra were fit with only the minimal constraints described above.

Error bars for Mössbauer measurements are discussed at length by Dyar [31] and Dyar et al. [38] for fits to well-resolved spectra studied here: ±0.02 mm/s for IS and QS and ±3% absolute on areas.

#### QEMSCAN analysis

QEMSCAN is an automated mineralogy solution method capable of investigating fine details of samples of interest. Using both energy-dispersive X-ray (EDX) spectra and backscattered-electron imagery (BEI) obtained via an x-y raster scan pattern [39], QEMSCAN measures mineralogic variability across a sample’s surface and can be used to quantify modal abundances of mineral species. Prior to each scan, instrument calibration is performed using the procedures outlined by Ayling [40].

Minerals are identified using a reference Species Identification Protocol (SIP), a hierarchical mineral database which determines what elements best fit a measured spectrum [41], [42]. For this investigation, the Oil and Gas (O&G) v. 3.3 SIP is used, with an accelerating voltage of 20 Kev and specimen current of ∼5 nA, and data are collected using iDiscover 5.2 beta software. A secondary SIP, Log5, also was used; however, it lacks detail in clay typing and therefore is less suitable for identification of verdine clays of interest.

Three representative polished and carbon-coated thin sections from the MGB (TX-18, TX-9 & TX-17) were analyzed using the O&G SIP. Total scan area was 3 by 3 mm, comprised of 9 blocks with sufficient overlap for easy stitching to produce a composite digital image. The resulting field images provide a 2-D view of the mineralogy of the sample illustrated in a color pattern from which quantitative modal mineral abundances can be extracted. Each analysis contains six false color digital images. The first image shows all the minerals detected, coded by various colors. The remaining five images characterize individual minerals textures and abundances. Pellets, grains and matrix are easily differentiated. Grain orientation, pellet size, shape, and abundance also are clearly apparent. QEMSCAN images were used to assist in selection of regions for analysis by electron microprobe.

## Results

### Petrography

#### Sediment

In a sedimentologic study of the central MGB, Zuschin and Stanton [9] recognized three microstratigraphic subunits: 1) homogeneous green minerals that they termed glauconitic siltstone, 2) bioturbated bioclastic, pelleted silty sandstone, 3) shell concentrations. Thin sections from the central MGB show bioclasts and pellets in a green clay matrix with quartz fragments. Area per cent, as determined by QEMSCAN, reveals sediment composed of clay pellets, as much as 50%, in a clay matrix. Quartz grains comprise up to 20%, and they are angular and poorly sorted. Shell fragments are a minor constituent, 2 to 4%. At top of the MGB, concretionary burrows contain varying amounts of clay pellets from 3 to 20%, with few quartz grains and shell fragments, less than 3%. Larger heterogeneous pellets (described below), are evident in the concretionary burrow fill.

#### Pellets

**Figure 8:**
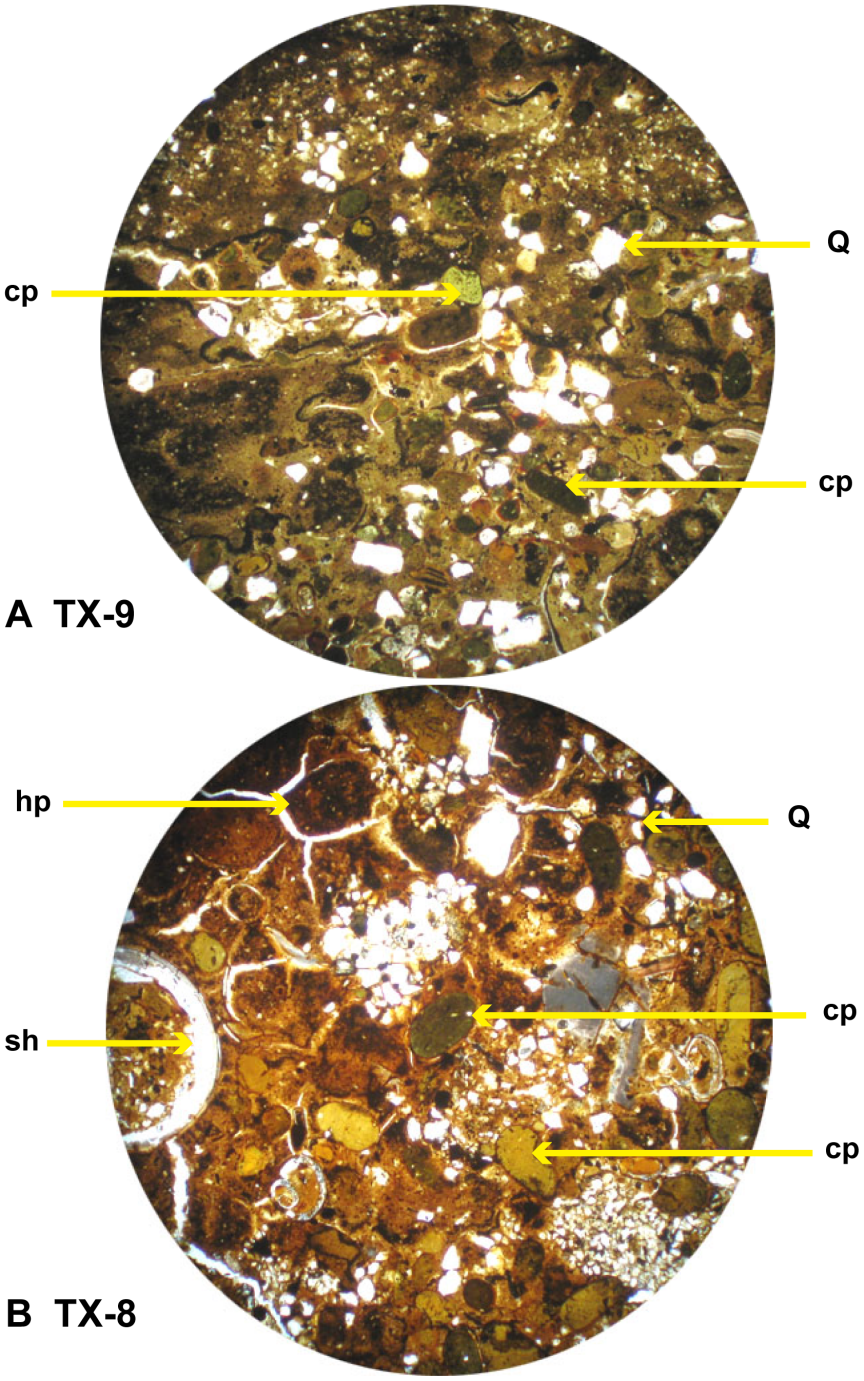
Photomicrographs from central MGB, A) TX-9, B) TX-8, cp = clay pellet, hp = heterogeneous pellet, sh = shell, Q = quartz.

**Figure 9:**
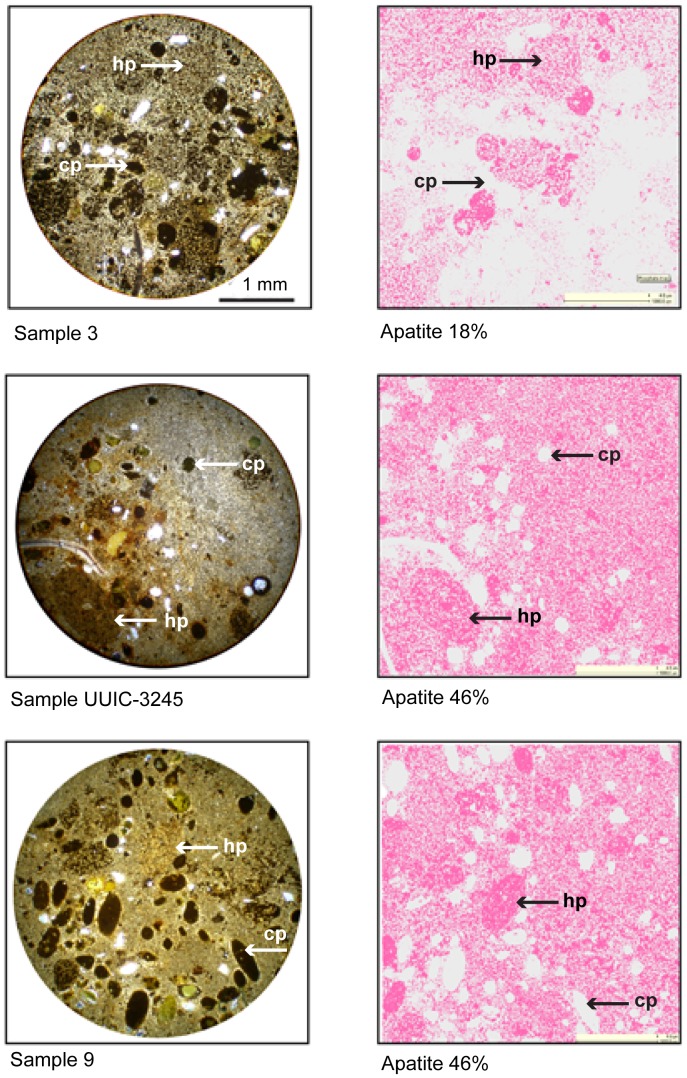
Top of MGB concretionary burrow fill, photomicrographs and QEMSCAN (false color) images, cp = clay pellets, hp = heterogeneous pellets, pink = apatite cement.

Two pellet types are recognized on the basis of size and composition: smaller clay pellets (cp) and larger heterogeneous pellets (hp). The clay pellets are green to black, tightly compacted ovoids that are easily observable with a hand lens. They are abundant in the central MGB (Figure 8). They also occur in varying amounts as burrow fill inside the concretions at top of the MGB (Figure 9). More obscure are the heterogeneous pellets that are found in both the central MGB and in burrows at the top of the MGB. In the concretionary burrow fill, the heterogeneous pellets were preferentially altered to apatite over siderite, which highlights their visibility, while the clay pellets remained unaltered. The two pellet types are displayed in photomicrographs of samples 3, UUIC-3245, and 9 top of the MGB with adjacent QEMSCAN false color images (Figure 9).

Abundance of clay pellets varies in the central MGB according to the three microstratigraphic subunits described above where they may comprise as much as 50 area %. At the top of the MGB within the concretionary burrows, clay pellets are scattered with some quartz and shell fragments in the matrix of siderite and apatite cement. The smallest clay pellets measure 0.2 mm length and 0.09 mm width, while the largest pellets average 1 mm length and 0.6 mm width. All are ovoid shape with some elongate ovoids. Clay pellets appear well indurated with sharp margins and smooth glossy surfaces. These pellets are thought to be of fecal origin based on their shape and the oxidation state of iron, which is chemically more reducing in the pellets compared to clay matrix. Very few pellets occur as internal filling of foraminifera tests. Angular quartz fragments occur primarily in the clay matrix and are not incorporated in the clay pellets. There is no evidence of internal canals within the clay pellets, as might be expected in decapod crustacean fecal pellets, such as *Palaxius* or *Favreina*. Although the most prominent burrows preserved in the MGB were excavated by decapod crustaceans, the fecal pellets in the sediment were produced by some other, presumably soft-bodied and unpreserved, animals.

The larger, heterogeneous pellets are not obvious in outcrop but are evident in thin section and in QEMSCAN images. Pellet shape in two-dimensional view is oval to circular, depending on thin section orientation. Pellets have sharp boundaries and consistent ovoid shape. Their size is consistently larger (average length 1 mm and width 0.6 mm) than the above-described green to black clay pellets. We see no evidence of diagenetic enlargement. These pellets are evident in the central bed and in the concretionary burrow fill at top of the MGB. In the central bed, microprobe and QEMSCAN analyses indicate that the heterogeneous pellet composition is dominated by clay minerals and tiny quartz fragments. At the top of the MGB, these pellets were altered to apatite and a lesser amount of siderite. Diagenetic processes did not alter or mask the original pellet shapes, and there is no evidence of internal canals. These larger, heterogeneous pellets indicate an alternate pellet producer. The pellet variety represents biological diversity in the MGB.

#### Color

The central MGB appears olive gray in outcrop. In thin section, the verdine clays appear green to brown. Stenzel [1] described the MGB as follows: “Olive-gray weathering to grayish-red, massive to poorly bedded, weathering to brownish-black, and grayish-red, sideritic calcareous concretions loosely spaced in the middle and crowded near the top to form a continuous clay ironstone layer, the bed forms a slight bench and a vertical face in the bluff.” Upon close examination, the color of the smaller clay pellets varies from several hues of green to brown and black. Munsell colors of the pellets include light olive brown (5Y 5/6), moderate olive brown (5Y 4/4), olive gray (5Y 3/2), moderate brown (5YR 3/4), brownish black (5YR 2/1), and olive black (5Y 2/1) [43]. Clay pellets commonly appear homogeneous, while larger pellets are mottled. Rims are sometimes lighter in color than centers. Color often is linked with authigenic mineral maturity or weathering, because the color of clay changes during diagenesis [16], [32], [34], [44].

### Mineral Composition

#### Clay

XRD, EMPA and Mössbauer spectroscopy are common methods used to identify clay minerals of the verdine facies [16], [24], [32]. Each was employed in this study, and the results were compared with published data. Since a 7Å reflection characterizes a number of clay types including odinite, kaolinite, serpentine, vermiculite, berthierine, chlorite or mixtures of clays, resolution of which clay types are the most likely required detailed evaluations.

Odinite, the dominant verdine clay type, was indicated based on its characteristics as first described by Bailey [24]. It is a dioctahedral-trioctahedral Fe^3+^-rich 1∶1 layer, 7.2Å clay mineral. It was published with the following structural formula which is based on sample #699: (Fe^3+^_0.78,_ Mg_0.77_ Al_0.56_ Fe^2+^_0.28_ Ti_0.02_ Mn_0.02_)_Σ = 2.42_ (Si_1.79_ Al_0.21_) O_5.00_ (OH)_4.00_ [24]. This formula includes a correction for the amount of SiO_2_ contamination (stated as ∼3%, but actually 2.51%), ignores measured water (using stoichiometric water instead), ignores measured alkali elements (Na, Ca, K), assumes an Fe^3+^/Fe^2+^ ratio measured on a different sample, and is normalized to 14 positive charges in the octahedral+tetrahedral sites. Recalculating the 699 analysis using the same total measured iron but fixing the Fe^3+^/Fe^2+^ ratio at 3.0 (X_Fe_ = 0.75), yields a nearly identical formula.

(Fe^3+^_0.79_ Mg_0.77_ Al_0.55_ Fe^2+^_0.26_ Ti_0.02_ Mn_0.01_)_ Σ = 2.42_ (Si_1.79_ Al_0.21_) O_5.00_ (OH)_4.00_.

**Figure 10:**
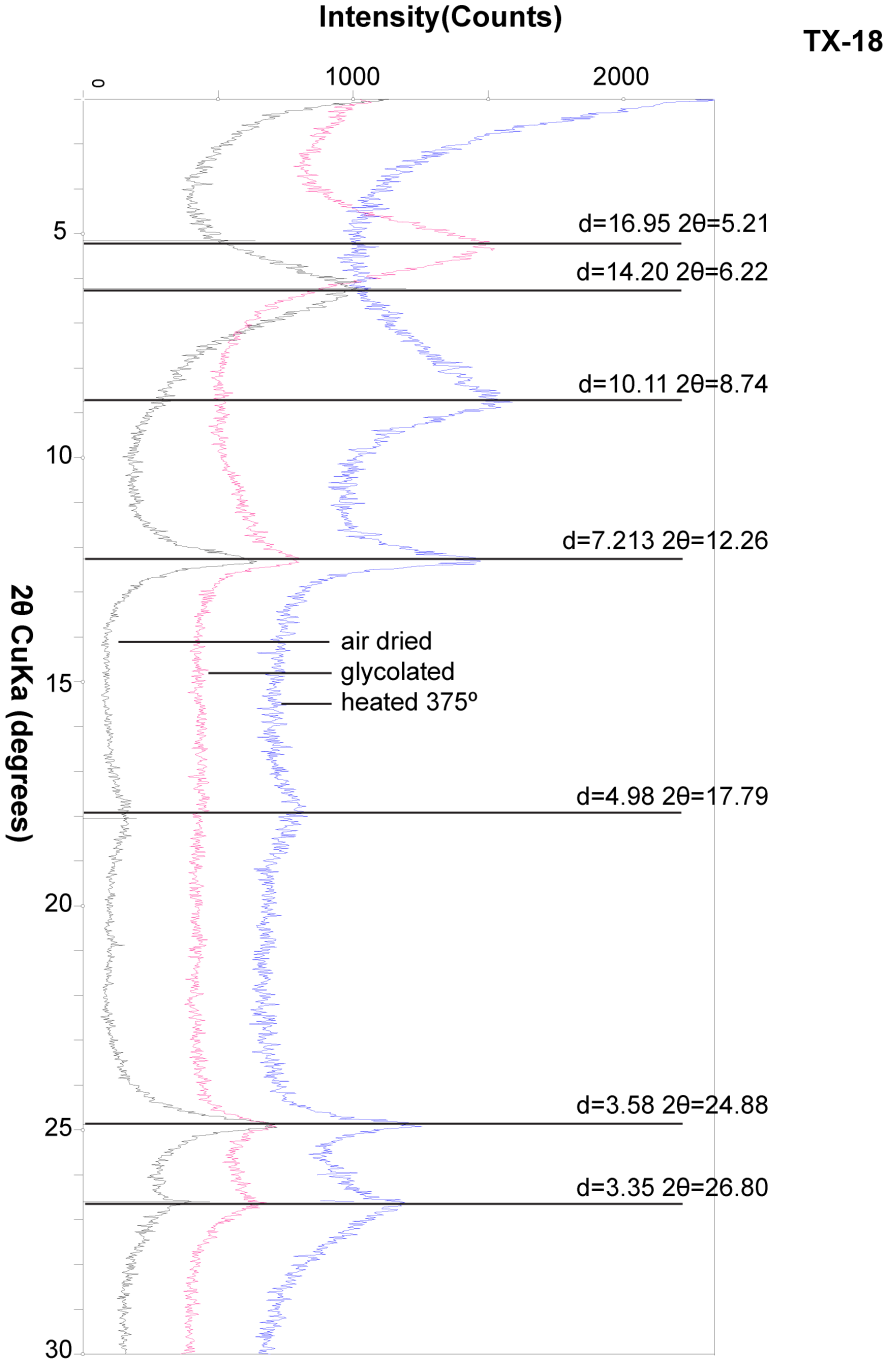
X-ray diffraction patterns of oriented, 2 µm clay fraction, from bulk sample TX-18, a mixture of 7 and 14Å clays (air dried, glycolated and heated to 375°).

X-ray diffraction analyses of both oriented and unoriented clay mounts consistently exhibited strong reflections at 7.2Å and 3.58Å. The oriented 2 µm clay, in a pellet-matrix mix from the central MGB, exhibited said reflections (Figure 10 and Figures S1 & S2). Another strong reflection occurs at 14Å in air-dried results. It is confirmed as smectite based on comparing diffraction patterns of air-dried and ethylene glycol treated samples where the 001 peak shifts to a very strong peak near 16.9Å. Illite is indicated by very weak 001, 002 and 003 reflections at 10.1, 5 & 3.34Å respectively. Kaolinite has reflections that coincide with odinite, but it was not confirmed here by other analyses. XRD patterns show no evidence for glauconitic minerals in the MGB. Peaks are not apparent at approximately 10.5 and 3.3, which would indicate glauconitic minerals.

**Figure 11:**
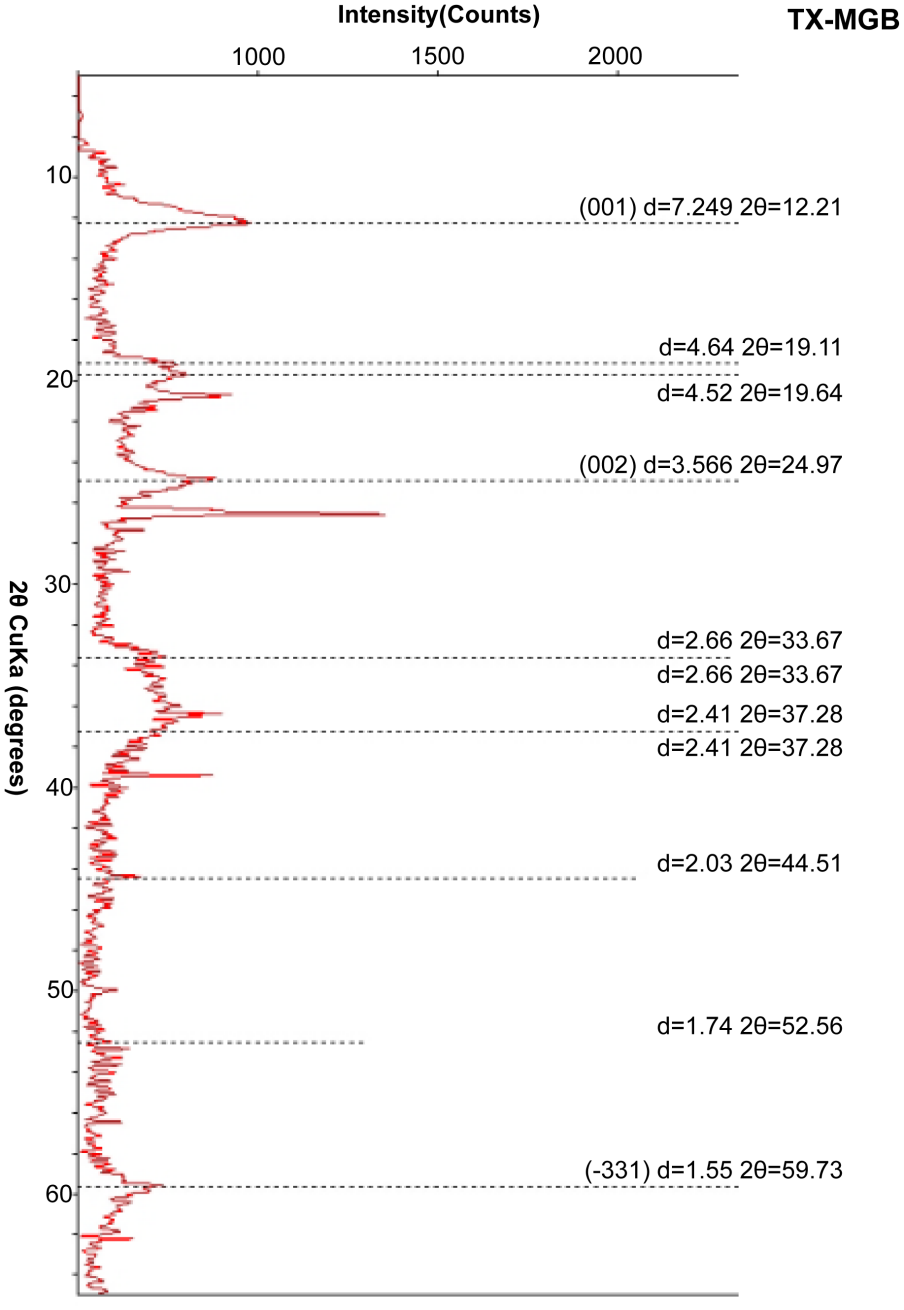
X-ray diffraction pattern of an unoriented powder mount of concentrated clay pellets, TX-MGB. Eleven labeled peaks were indexed in unit cell calculation for odinite-1M. Unlabeled peaks are quartz. Peak at 2θ = 59.73 is quartz overlapped by odinite.

Random oriented powders from MGB clay pellet separates yielded peaks at 7.249Å and 3.566Å (001 and 002) (Figure 11). The odinite-1M data base peaks are very similar at 7.150Å and 3.580Å [45]. Eleven peaks were indexed and compared with the odinite-1M data base in unit cell calculation (Table 1). This refinement process indicates that odinite is a reliable interpretation for the 7.2Å clay peaks that appear in all the x-ray diffractograms in this study.

**Table 1:** Unit cell calculation from XRD data (odinite-1M (monoclinic)).

	Sample TX-MGB	SD	PDF# 00-048-1857	SD
a =	5.35	±0.01	5.38	±0.01
b =	9.38	±0.03	9.34	±0.02
c =	7.42	±0.03	7.39	±0.02
Beta =	104.79	±0.40	104.03	±0.25

Eleven peaks were indexed. MGB pellets compared with ICDD. All units are in angstroms (Å).

X-ray diffraction analyses of randomly oriented powders, in a pellet-matrix mix, in the location of 060 were evaluated. In analyses of verdine facies minerals, Odin [16] found wide and asymmetric peaks between 1.56Å (59.23° 2θ) and 1.51Å (61.35° 2θ) in the vicinity of the 060 diffraction peak. This peak gradually decreases towards 1.50Å. Results for the three central MGB samples, comprised of pellets and clay matrix, show the 121- (*hkl*) peak of quartz at 1.54Å (59.970° 2θ) [46] which partially masks the reflections for verdine minerals. The broad peak, described by Odin [16] is subtle but present in MGB diffractograms which indicates the presence of verdine minerals (Figure S3). A 060 diffraction peak for kaolinite (62.31° 2θ) [33] is not present which is consistent with EMPA and QEMSCAN results that show a lack of kaolinite in MGB samples.

**Figure 12:**
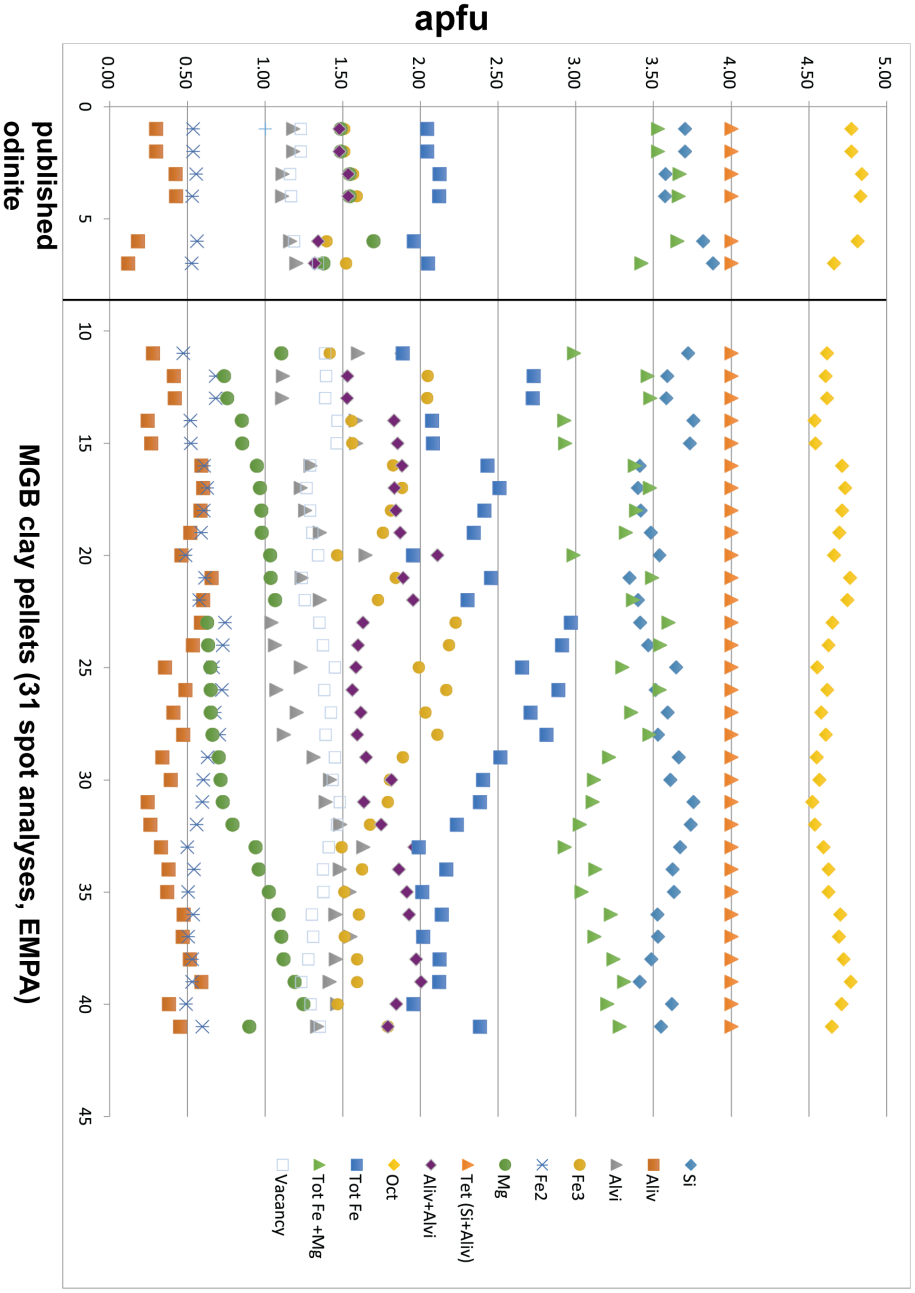
Chemical analyses (apfu), published [16], [24] and MGB clay pellets, used to calculate mineral formula.

The electron microprobe results of 31 EMPA spot analyses of MGB clay pellets show consistent composition among the important cations, Representative EMPA analyses are given in Table 2. The maximum, minimum and mean of the 31 analyses are compared with published results (Table 3). The data were converted to apfu (atoms per formula unit), and they are illustrated adjacent to samples #699 and #508 (odinite in the type locality) [16], [24] and presented in Figure 12. The comparison shows fairly consistent apfu results for the important cations. The formula calculated herein follows Bailey’s [24] procedure but fixes the atomic Fe^3+^/Fe^2+^ ratio at exactly 3.0. The following average structural formula calculated for MGB clay pellets is: Fe^3+^_0.89_ Mg_0.45_ Al_0.67_ Fe^2+^_0.30_ Ti_0.01_ Mn_0.01_)_ Σ = 2.33_ (Si_1.77_ Al_0.23_) O_5.00_ (OH)_4.00_. Odin’s formula for #699 is slightly more Mg rich, and Fe, Al poor. This identifies odinite as the dominant clay mineral in MGB clay pellets. Raw chemical data for odinite analyses in the MGB clay pellets are presented in Table S1.

**Table 2:** Representative analyses from MGB clay pellets, 11 each from the central MGB and concretionary burrow fill at the top of the MGB.

	Central MGB ––>				Top MGB ––>				
sample #	TX-8	TX-18	TX-18	5c	9	9	W	TX-17	TX-17
**n** *	6	2	2	1	3	1	1	2	4
**SiO2**	33.9	33.2	35.5	33.9	33.7	34.4	34.8	35.7	32.8
**Al2O3**	15.8	12.0	14.8	14.5	13.2	12.7	13.1	13.4	15.4
**Fe2O3T**	31.8	33.6	26.1	22.8	37.4	33.2	38.1	31.3	25.5
***Fe2O3***	*23.8*	*25.2*	*19.6*	*17.1*	*28.0*	*24.9*	*28.5*	*23.5*	*19.1*
***FeO***	*7.15*	*7.55*	*5.88*	*5.13*	*8.41*	*7.48*	*8.56*	*7.05*	*5.74*
**MgO**	6.63	4.63	5.41	6.73	4.17	4.09	4.31	4.60	6.72
**MnO**	0.18	0.10	0.06	0.10	0.06	0.07	0.04	0.08	0.15
**TiO2**	0.30	0.24	0.24	0.22	0.16	0.20	0.07	0.23	0.21
**CaO**	1.03	1.12	1.37	0.42	0.84	0.85	0.36	0.87	0.55
**Na2O**	0.08	0.05	0.06	0.10	0.10	0.09	0.14	0.06	0.09
**K2O**	0.34	0.50	0.63	0.90	1.12	1.76	1.45	0.78	0.46
**H2O**	11.9	11.1	11.4	10.9	11.6	11.3	11.9	11.6	11.1
**F**	0.03	0.52	0.19	0.36	0.02	0.16	0.00	0.41	0.23
**Cl**	0.06	0.02	0.07	0.14	0.30	0.00	0.18	0.01	0.19
**Sum with FeO+Fe2O3**	**101.2**	**96.3**	**95.1**	**90.5**	**101.8**	**98.0**	**103.5**	**98.3**	**92.8**

* Number of spot analyses in one pellet.

**Table 3:** Maximum, minimum, mean and standard deviation of chemical analyses from MGB pellets and published odinite.

	MGB pellets				Odinite (samples 699 & 508)			
*Oxide Wt %*	Min	Max	Mean	SD	Min	Max	Mean	SD
			n = 31				n = 6	
**SiO2**	28.0	36.1	33.6	1.62	33.5	37.5	35.6	1.71
**Al2O3**	11.5	16.5	14.4	1.41	11.0	12.2	11.8	0.67
**Fe2O3T**	22.8	38.2	30.1	4.57	25.1	26.4	26.2	0.51
**Fe2O3**	*17.1*	*28.6*	*22.6*	*3.43*	*17.9*	*19.5*	*20.4*	*3.01*
**FeO**	*5.13*	*8.59*	*6.76*	*1.03*	*6.10*	*6.50*	*6.25*	*0.15*
**MgO**	3.95	7.90	5.71	1.16	9.70	11.0	9.78	0.68
**MnO**	0.04	0.31	0.14	0.07	0.00	0.33	0.22	0.17
**TiO2**	0.07	0.62	0.22	0.11	0.40	0.50	0.35	0.18
**CaO**	0.36	1.37	0.88	0.27	0.13	0.50	0.22	0.15
**Na2O**	0.02	0.16	0.08	0.04	0.00	0.20	0.07	0.10
**K2O**	0.26	1.76	0.71	0.40	0.35	1.30	0.63	0.44
**H2O**	10.5	12.0	11.4	0.41	10.0	15.0	11.6	1.81
**F**	0.00	0.82	0.20	0.18				
**Cl**	0.00	0.60	0.09	0.12				
**Sum** *	**76.9**	**115.3**	**96.7**		**89.1**	**104.6**	**96.8**	

* Sum with FeO+Fe2O3.

Glauconitic mineral grains, although small and very sparse, were the focus of some spot analyses in an attempt to locate a potassium X-ray signal. The glauconitic mineral analyses (>2 wt.% K_2_O) were omitted from the above structural formula calculation for odinite. MGB clay pellets, for the most part, are low in K_2_O, with an average of 1.5 wt. %. The few glauconitic grains encountered were small and fractured. Three analyses showed K_2_O >6 wt. %, which qualifies as mature glauconite. Thus, the Main Glauconite Bed actually contains very little glauconite (3% by QEMSCAN). Raw data for glauconitic minerals and other clays are presented in Table S2.

**Figure 13:**
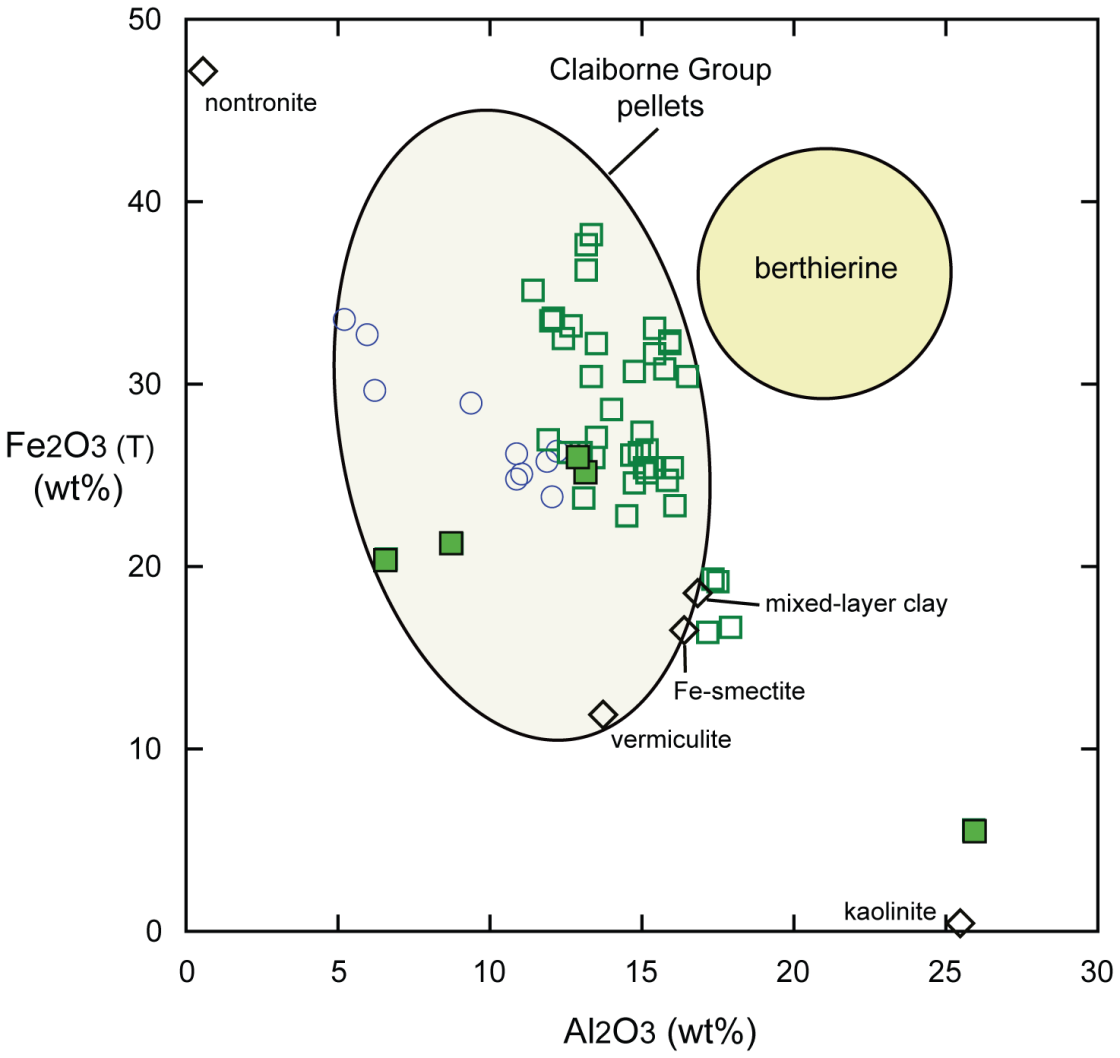
MGB clays plotted within the compositional field of iron-rich clays from the Claiborne Group. Open squares (green) are from the MGB and solid squares are glauconitic minerals from the MGB. Open circles are published analyses of odinite [16], [24]. The range of compositions of clay in pellets from the Claiborne Group is indicated by the large oval [11], [61]. The field of berthierine is from Brindley [61].

Several clay types were characterized in a study of Claiborne Group iron-rich authigenic clays by Huggett et al. [11], [21]. They used the general term serpentine for 7Å iron-rich mixed layer clays, including odinite. The average chemistry of clay pellets from the MGB is very similar to the average of 53 analyses for pellets from the upper 8 m of the Crockett Formation, which includes the MGB, from the same locality as this study [11]. All MGB clay pellet analyses from this study, including glauconitic grains, are displayed as total Fe_2_O_3_ plotted against Al_2_O_3_ together with the published results (Figure13). The compositional variation of MGB clay pellets is evident, where they are seen to lie within the compositional field of authigenic iron-rich clays from other Claiborne Group clays [11]. Also shown are published analyses of other clays including nontronite, berthierine, Fe-smectite, vermiculite and kaolinite. These, along with the MGB clay pellets, tend to lie along a general trend between nontronite and kaolinite, and are distinctly lower in Al_2_O_3_ than berthierine (Figure 13) [11].

**Figure 14:**
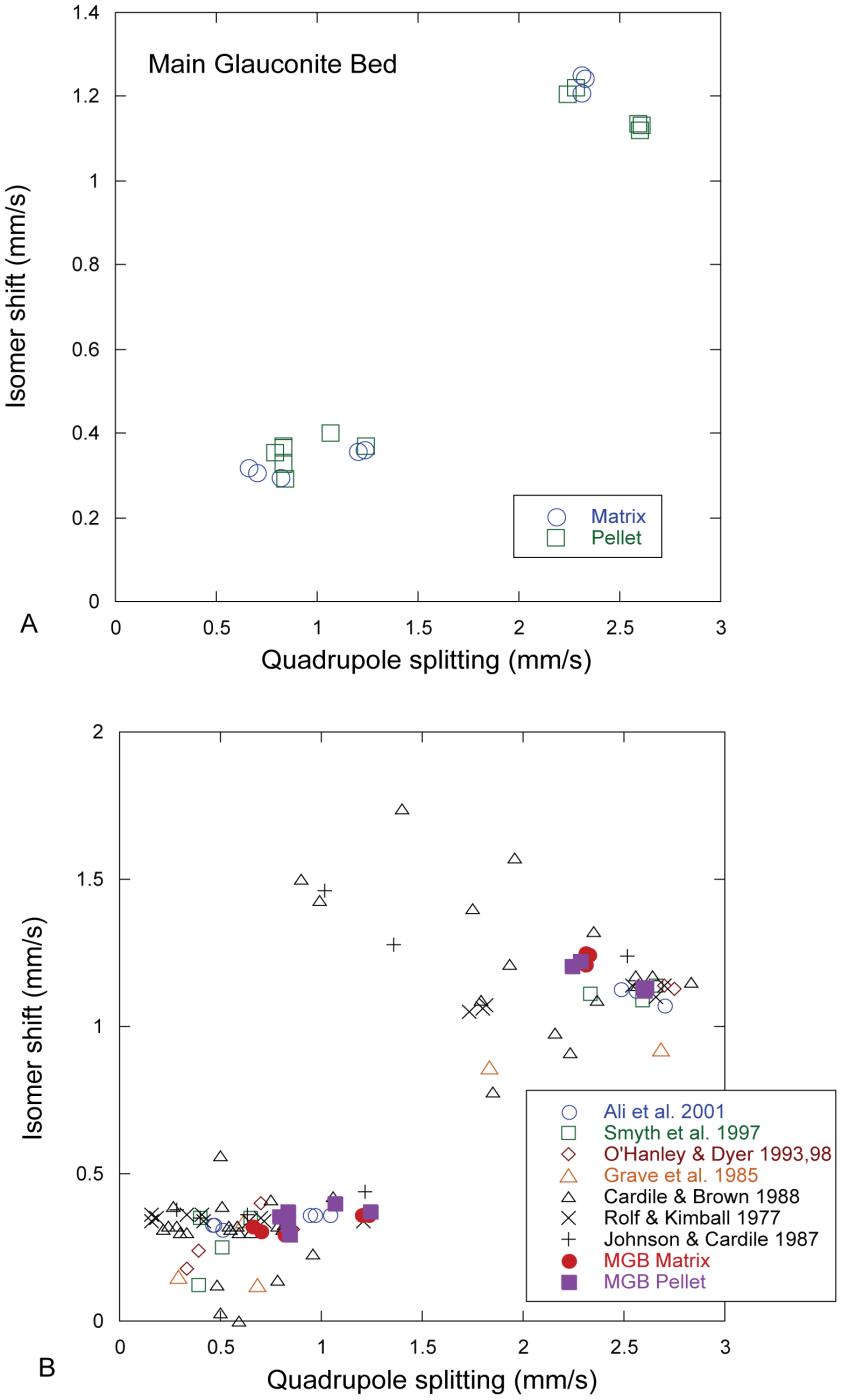
Plot of isomer shift vs. quadrupole splitting (in mm/s). A) Clays from the MGB (this study). B) MGB clays compared to published analyses of glauconite.

Mössbauer spectra were obtained on clay separates from the rock matrix and pellets (Figure S4), and Mössbauer parameters are provided in Table 4. Peak area ratios indicate a predominance of Fe^3+^ (63–81% of total Fe). Ferric/ferrous ratios in clays in fecal pellets are lower than in matrix clays from the same sample, indicating that the biogenic nature of the pellets provides micro-reducing environments on the seafloor favorable for the formation of verdine minerals. Figure 14 shows a plot of isomer shift vs. quadrupole splitting for the MGB clays together with representative published data for glauconite. For the MGB clays Fe^3+^ values of the isomer shift fall between 0.30 and 0.40. Of the eight samples, four had doublet pairs for Fe^3+^ resulting in a bimodal distribution in QS of 1.02–1.24 and 0.66–0.84. In the case of the other four samples in which there was a single doublet for Fe^3+^, the range in values for QS falls within the lower range, 0.70–0.83.

**Table 4:** Mössbauer parameters of 7Å clay mixture.

Sample		5c	5c	TX-9	TX-9	TX-18	W	W	W
		matrix	pellets	matrix	pellets	pellets	matrix	m & p	pellets
**ferric**	**IS**	0.31	0.36	0.30	0.36	0.33	0.32	0.29	0.37
	**QS**	0.70	0.83	0.82	0.79	0.83	0.66	0.84	0.83
	**Width**	0.49	0.62	0.34	0.33	0.54	0.42	0.38	0.59
	**Area %**	81.3	66.4	46.3	30.2	77.1	65.8	39.0	68.3
**ferric**	**IS**			0.36	0.40		0.36	0.37	
	**QS**			1.20	1.07		1.23	1.24	
	**Width**			0.43	0.59		0.38	0.45	
	**Area %**			26.2	33.0		14.4	28.7	
**ferrous**	**IS**	1.21	1.13	1.25	1.12	1.21	1.25	1.22	1.14
	**QS**	2.31	2.61	2.33	2.60	2.25	2.31	2.28	2.59
	**Width**	0.40	0.22	0.26	0.32	0.30*	0.40*	0.30*	0.30*
	**Area %**	18.7	33.6	27.5	36.8	22.9	19.8	32.3	31.7
	**% Fe^3+^**	**81**	**66**	**72**	**63**	**77**	**80**	**68**	**68**
	**% Fe^2+^**	**19**	**34**	**28**	**37**	**23**	**20**	**32**	**32**

IS: Isomer shift (mm/s).

QS: Quadrupole splitting (mm/s).

Width: FWHM (mm/s).

Spectrum temperature 295 K.

* parameter fixed.

Collectively, these data suggest the presence of two slightly different octahedral sites for Fe^3+^, possibly suggesting some trioctahedral character in the clays. The difference in the average of the quadrupole splitting between the two sites, 0.41 mm/s, is similar to that reported for glauconite by Ali [47] but shifted to higher values by about 0.25 mm/s. Based on peak areas (Table 4), Fe^3+^ is more abundant in the site characterized by lower quadrupole splitting. The isomer shift for Fe^2+^ ranges from 1.13–1.25 mm/s, and the data fall into two clusters, especially defined by QS. There were no doublet pairs fit to the Fe^2+^ spectra. Nonetheless, the distinct bi-modal array of IS and QS values suggests again the presence of two octahedral sites for Fe^2+^. Because of its substantial Fe content, there is considerable literature on Mössbauer spectra of glauconite, and a number of workers have allocated Fe to *cis*-M2 and *trans*-M1 sites [47]–[51]. However, Rancourt and co-workers [52]–[54] have argued that these sites may not be positively delineated, and that the spectral data may be recording local distortion environments. Dyar [55] suggested that hydrogen content may be responsible for variation around octahedral sites because of the difference in the location of hydroxyls around M1 and M2 sites. We do not have direct determination of H_2_O contents of MGB clays, but they do have measureable F and Cl, and both vary in concentration. Substitution of F and Cl, and possibly O in OH sites could change the geometry of the adjacent *trans* and *cis* sites and be responsible for the bimodal behavior of MGB clays.

The Mössbauer Fe^3+^/Fe^2+^ ratio is diagnostic for identifying verdine clay minerals. The average Fe^3+^/Fe^2+^ ratio in MGB samples of matrix and pellets is 73∶27%. A similar ratio of 73.6∶26.4% was calculated for phyllite-V clay (later named odinite) in modern sea- floor clays [16]. The Fe^3+^/Fe^2+^ ratio of clay matrix is slightly higher than that of clay pellets: 78∶23%, versus 68.5∶31.5%, respectively. The biogenic nature of the pellets provides micro-reducing environments on the seafloor favorable for the formation of verdine minerals.

QEMSCAN reports chemical data according to the SIP mineral definition. The O&G SIP tests for clays in the following order: glauconite, illite, kaolinite, nontronite, smectite and chlorite. Odinite is not defined in the O&G SIP algorithm, but its composition strongly overlaps with chlorite, and odinite is known to alter to chlorite [24]. Therefore, the O&G SIP was adjusted to accommodate the MGB clay samples by changing the mineral name from chlorite to odinite for this study. During QEMSCAN analysis of MGB clays, some 50% ended up in chlorite groups by default and are regarded as odinite.

**Figure 15:**
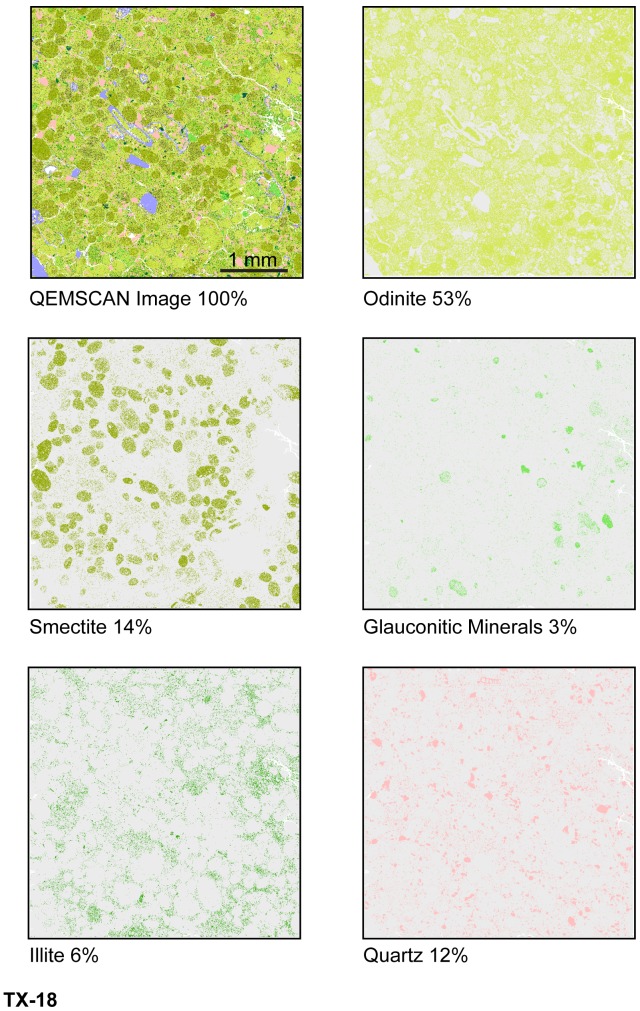
Central MGB QEMSCAN images with specific mineral area %, sample TX-18.

**Figure 16:**
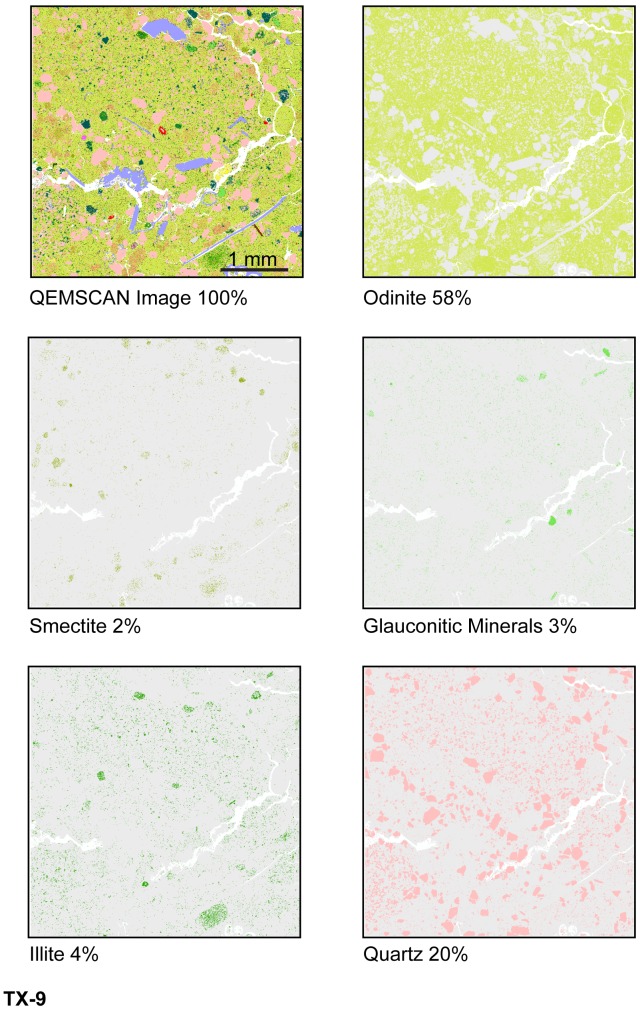
Central MGB QEMSCAN images with specific mineral area %, sample TX-9.

**Figure 17:**
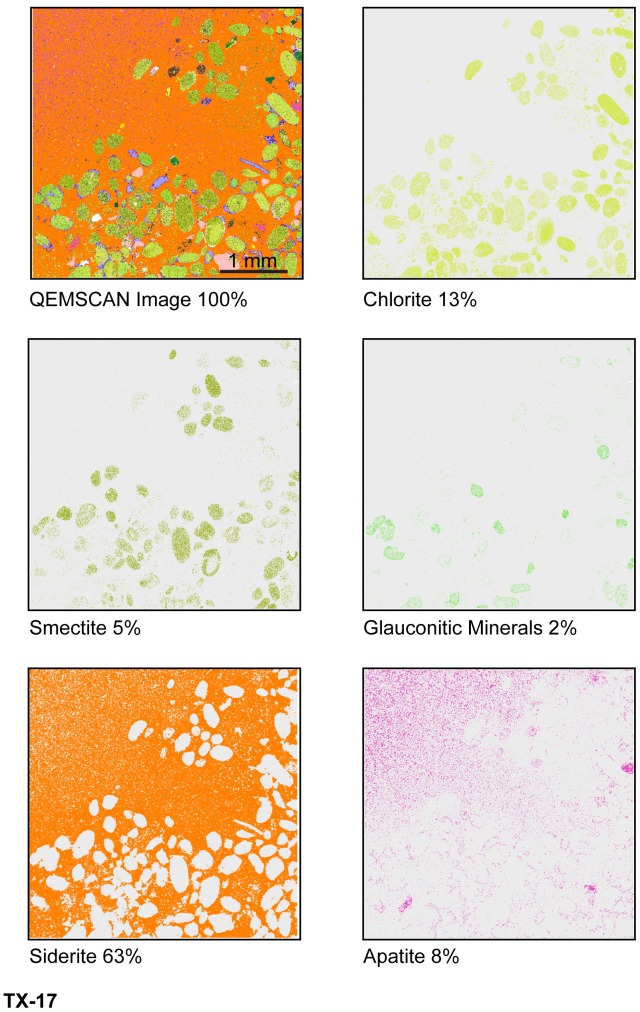
Top of MGB QEMSCAN images with specific mineral area %, sample TX-17.

Odinite and small amounts of smectite, illite and glauconitic minerals (<3 area %) are displayed in various shades of green in digital false color on QEMSCAN textural maps (Figures 15, 16, 17). Textural and compositional variation in the central MGB (Figure15) shows that clay matrix is predominantly odinite and illite, while clay pellets are predominantly odinite and smectite. Figure 16 demonstrates that odinite is dominant in both the matrix and clay pellets with minor amounts of illite, smectite and glauconitic minerals. Glauconitic minerals occur in fairly pure grains, although very few in number (about 3 area %). QEMSCAN analyses from the central MGB show that quartz grains average 18 area % and calcite averages about 3 area %, occurring as shell fragments.

Concretionary burrow fill at the top of the MGB is predominantly siderite and apatite cement, in various quantities. Sample UUIC 3245 had 46∶43 area % apatite-siderite, while sample 9 had 46∶39 apatite-siderite. Other concretionary burrows are dominated by siderite. Textural map, Figure 17, shows an 8∶1 mixture siderite-apatite. False color pink represents the P_2_O_5_-rich mineral, apatite. Clay pellets remain unaltered even though they are incorporated in the concretionary burrow fill.

The identification of odinite, a key verdine mineral, is supported by all of the above analytical results. The unit cell calculation shows lattice parameters that are uniquely odinite. It is the dominant clay type in the MGB clay pellets. The structural formula calculated from EMPA data is consistent with that of odinite. X-ray diffraction results suggest an odinite, smectite, illite clay mixture. Verdine facies clays besides odinite include smectite and Fe^3+^ chlorite, sometimes referred to as swelling chlorite [16], [24]. The MGB clay is moderately high in Fe, and Mössbauer spectroscopy found an approximate Fe^3+^/Fe^2+^ ratio of 3∶1, which is consistent with odinite. QEMSCAN identified the dominate clay type of the MGB as chlorite-like, which has close similarity to odinite.

#### Siderite and apatite

Concretions at the top of the MGB are composed of a fine-grained apatite-silicate mixture. Analyses of siderite are presented in Table 5. Despite attempts with the electron microprobe to locate regions without a phosphorus X-ray signal, when measured, phosphorus was always detected, ranging in three samples from 0.8 to 4.0%, which corresponds to about 2–10% apatite. A small amount of Al_2_O_3_ was also recorded, and probably represents a minor amount of clay. Mole percent carbonate end-members are calculated with CaCO_3_ adjusted for the Ca-equivalent of apatite as determined from P_2_O_5_ concentrations when measured (Table 5). Mole fraction FeCO_3_ ranges from 70–75%, consistent with the observation of Mozley [56] that marine siderite has <95 atom% Fe and that Mg>Ca. Huggett et al. [11] report a slightly higher cation mole fraction of 0.79 for siderite from the MGB from a partial analysis.

**Table 5:** Concretionary burrow fill, MGB top, siderite and mole percent carbonate end-members calculated with CaCO_3_.

Sample	3	9	3245	1 plug 4	1 plug 8	Avg	SD
**n** *	1	3	1	1	1	7	
**SiO_2_**	2.30	2.50	1.90	1.16	2.42	2.05	0.55
**TiO_2_**	0.00	0.05	0.03	0.06	0.04	0.03	0.02
**Al_2_O_3_**	1.60	0.90	1.00	0.50	1.14	1.01	0.40
**FeO** **	37.3	41.3	43.2	38.7	38.2	39.7	2.20
**MnO**	0.74	0.65	0.62	1.02	0.75	0.76	0.16
**MgO**	6.00	4.76	4.90	4.63	5.23	5.10	0.55
**CaO**	4.35	8.81	7.45	4.65	3.89	5.83	1.94
**Na_2_O**	0.17	0.15	0.06	0.12	0.20	0.14	0.06
**K_2_O**	0.21	0.08	0.06	0.05	0.19	0.12	0.08
**P_2_O_5_**	0.76	4.04	2.66				
**Total**	53.4	63.3	61.9	50.9	52.0	54.7	5.3
**% apatite**	1.8	9.6	6.3				
**Mol %**							
**CaCO_3_** ***	8.1	8.2	8.8	11.0	9.4	13.1	2.0
**MgCO_3_**	20.2	15.4	15.2	15.3	17.5	15.9	2.0
**MnCO_3_**	1.4	1.2	1.1	1.9	1.4	1.4	0.3
**FeCO_3_**	70.3	75.2	75.0	71.7	71.7	69.6	2.3

* Spot analyses in one grain.

** Total Fe as FeO.

*** CaCO_3_ corrected for apatite.

Analyses of concretions consisting of a fine-grained mixture of apatite and one or more alumino-silicates are provided in Table 6. Assuming that all P is contained in apatite, and accounting for the equivalent CaO, the composition of the silicate component (neglecting halogens) can be estimated. The calculated silicate component is similar in composition to MGB clays with respect to total Fe_2_O_3_ and Al_2_O_3_, although with slightly lower SiO_2,_ suggesting that the silicate in the phosphate-bearing cement is the same or very similar to the clay in the pellets and the matrix of the MGB.

**Table 6:** Concretionary burrow fill, MGB top, a fine-grained mixture of apatite and one or more alumino-silicates.

Sample	3	3	9	3245	Avg	SD
**n** *	1	2	2	2	7	
**SiO_2_**	11.6	17.0	11.3	16.5	14.1	3.07
**TiO_2_**	0.20	0.19	0.15	0.17	0.18	0.02
**Al_2_O_3_**	5.60	8.20	5.71	8.97	7.12	1.72
**Fe_2_O_3_** **	15.7	13.6	12.1	15.9	14.3	1.82
**MnO**	0.19	0.07	0.09	0.07	0.11	0.06
**MgO**	1.68	1.61	0.89	1.56	1.43	0.37
**CaO**	26.9	25.2	34.4	27.8	28.6	4.05
**Na_2_O**	0.35	0.41	0.46	0.30	0.38	0.07
**K_2_O**	0.34	0.45	0.31	0.47	0.39	0.08
**P_2_O_5_**	19.8	18.6	23.9	19.4	20.4	2.36
**F**	1.96	1.90	0.01	0.02	0.97	1.10
**Cl**	0.03	0.03	2.36	2.00	1.11	1.25
**sum**	84.2	87.3	91.7	93.2	89.1	4.12
**Less O = F,Cl**	0.83	0.81	0.54	0.46	0.66	0.19
**Total**	**83.4**	**86.5**	**91.2**	**92.7**	**88.5**	**4.29**
**% apatite**	46.8	44.0	56.5	45.9	48.2	4.9

* Spot analyses in one grain.

** Total Fe as Fe_2_O_3._

## Discussion

The detailed mineralogy and geochemistry of the MGB determined in this study relate to paleoecology, sedimentology and stratigraphic findings from previous studies. When integrated with the ichnology of the MGB, a coordinated approach to understanding the depositional environment in a sequence stratigraphic context is possible. Verdine minerals, specifically odinite, were identified in the clay pellets. Thornton, in a 1994 master’s thesis [10], also noted that the MGB clays had the characteristics of odinite. Whether the MGB is comprised of odinite or authigenic verdine minerals that were altered, the paleoenvironmental implications are the same. It is presumed that the present-day verdinization environment is key to understanding the Middle Eocene continental shelf green marine clay environments.

### Pelleted Component

The mineralogy of the clay pellets and their occurrence in the central MGB and in concretionary burrow fill at the top of the MGB has implications for understanding depositional processes. Odinite-rich clay pellets are abundant in the central MGB. They are interpreted as fecal pellets based on their consistent shape, varied size, appearance and iron oxidation state. Their varied size is attributed to multiple producers, which reflects a diverse faunal community. Fecal pellets originally must have contained organic matter, resulting in locally more reducing conditions than the surrounding environment [24]. The pellets provided a favorable site for verdinization, because verdine minerals tend to form within sheltered, reducing, granular microenvironments. Clay pellets of the MGB have a lower Fe^3+^/Fe^2+^ ratio than the clay matrix (Table 4), and consequently they reflect more reducing conditions, as would be expected for fecal material.

Clay pellets occur in both the central bed and in the concretionary burrow fill at top of the MGB. In the central bed, pellets are not evenly distributed throughout, suggesting that they may have been reworked by gentle winnowing and episodic storms. Clay pellets at the top of the MGB could have been introduced into the open burrows along with other detritus, possibly reworked from the central MGB, or they could have been left behind by occupants in the burrow fill. The clay pellets do not show characteristics of those produced by decapod crustacean burrowers.

The larger, heterogeneous pellets composed primarily of the apatite-cementing agent, can be seen in both the photomicrographs and the QEMSCAN mineral images of concretionary burrow fill. In comparison with the clay pellets, the apatite pellets are larger and more mineralogically heterogeneous (Figure 9). These pellets also occur in the central MGB as a loosely compacted unaltered mixture of clay and tiny clasts. At top of the MGB, these pellets were incorporated into the apatite and siderite-cementing agents of the concretionary burrow fill. Based on their affinity for phosphate minerals and their shape, they are interpreted as fecal in origin. Like the clay pellets, the apatite pellets could have been left behind by occupants in the burrow fill. They do not show the morphologic characteristics typical of pellets produced by decapod crustacean burrowers.

A very few fragmented glauconitic mineral grains (3 area %) occur in both the central and top of the MGB. Their existence is confirmed by electron microprobe on the basis of K_2_O >2 wt. % and by QEMSCAN analyses based on SIP definition. The fragmented nature of the grains lends support to the hypothesis that some winnowing and reworking of sediments may have occurred, and that glauconitic minerals were transported from deeper environments where they originated. Glauconitic minerals, once formed, are very stable in the marine environment, and they may well survive transport of appreciable distance. The glauconitic grains may be more brittle than the non-glauconitic clay pellets, and so they tend to fracture more readily. Glauconitic grains found in the concretions were likely added with clastics in the burrow fill.

### Sequence Stratigraphy

In a sequence stratigraphic context, green minerals often are associated with condensed sections and transgressive-systems-tracts. A transgressive phase on a sediment-starved sea floor offers favorable conditions for the glauconitization or verdinization process. A regressive phase, on the other hand, may introduce a more energetic, oxidizing environment of higher sediment influx, which would inhibit clay authigenesis [25], [29]. Green minerals of the verdine facies suggest the shallow inner shelf, more proximal zone of the transgressive surface and transgressive-systems-tract.

The MGB is part of a regionally mapped transgressive sequence during the Middle Eocene in the northwest Gulf of Mexico. The siliciclastic depositional center was associated with the Houston Embayment (Figure 1) [57]. Deposition occurred during an interval of fluctuating sea level in sediment-starved conditions, interfingering with episodes of higher sedimentation rate. The basal contact of the MGB is sharp, irregular and burrowed. The central MGB has verdine facies clays that indicate shallow marine conditions with transgressive characteristics in proximity to river mouth influx (Figure 7). The upper contact of the MGB marks a boundary between two contrasting facies. It is irregular due to burrowing [2], [13]. The burrows are interpreted as firmground trace fossils that often occur in temporarily dewatered and compacted sediment. The top of the MGB may highlight a coplanar transgressive surface–sequence boundary in a parasequence. It is possible that the top of the MGB could have been exposed in an intertidal environment. Then the open burrows were filled with detritus during renewed submergence. Subsequent apatite and siderite cementation of the firmground would have occurred in a subaqueous setting.

The Crockett Formation was deposited in a complex marginal marine environment with dynamic sea level fluctuation. It represents a shallow-water transition zone in an overall upward deepening sequence. While odinite formed *in situ* associated with the fecal pellets of the central MGB, the few glauconitic mineral grains in the MGB may be explained by detrital reworking. Grains can be transported widely in transgressive systems, and glauconite is stable geochemically in the marine environment.

### Paleoenvironment

The paucity of glauconitic minerals indicates that seafloor conditions during deposition of the MGB were unsuitable for their formation, or that the glauconitization process was incomplete or arrested. This might mean a number of things. For example, perhaps sea-floor conditions were not consistently marine, too close to river mouth influx, too close to shore, or too shallow water depth. Sediment influx can inhibit the glauconitization process by not allowing enough time for glauconitic minerals to mature. The few, small glauconitic mineral grains in the MGB were often fractured, which indicates they may be of allochthonous origin, so it appears that the glauconitization process did not occur during deposition of the MGB.

On the other hand, verdinization apparently did occur. The presence of odinite, especially in the clay pellets, indicates a set of sea-floor environmental conditions pertaining to geochemistry, temperature, oxidation state, energy and latitude. The central MGB reflects depositional processes in a low energy, somewhat sheltered, shallow, warm, mostly marine environment. Sedimentation rate was sufficiently slow in proximity of continental input of iron and gentle winnowing currents to provide Eh and pH conditions suitable for verdine mineral authigenesis [16], [24], [32].

The change in mineralogy from the central bed to the top of bed indicates a change in the sea-floor environment. The central MGB was deposited in a near shore, subaqueous setting, while it is possible that the top of the MGB, with its concretionary burrow fill comprised of pellets and detritus, may have been in an intertidal environment. This interpretation is supported by characteristic burrowing patterns at top of the MGB. It represents an interval of non-deposition, dewatering, compaction and burrowing. Ultimately, the open burrows excavated by infaunal crustaceans were filled with sediment and bioclasts, indicating return to a subaqueous environment in moderate energy where precipitation of siderite and apatite cementing agents occurred.

A coordinated approach to understanding the paleoenvironment of the MGB incorporates verdine facie characteristics, ichnological signature, and findings from previous MGB studies. During a period of regional transgression, relatively high sea level, and some lateral continuity of facies, several coastal siliciclastic environments may apply. These include protected shoreface settings, large scale normal marine lagoons, interdeltaic bays, sounds, or embayments. Tropical seagrass beds demonstrate high molluscan diversity, such as that observed in the MGB. Seagrasses were present in the Gulf and Caribbean during the warmer climatic conditions of Middle Eocene [58]. Seagrass environments are recognized only rarely in the geological record due to the low preservation potential of soft plants, but they probably were much more abundant than is normally realized. Benthic foraminiferal evidence would be instructive, however that was outside the scope of this study. In general, the setting of the MGB is characterized as shallow inner shelf, proximal to some terrestrial influence.

## Conclusion

The MGB clays at Stone City Bluff of Middle Eocene, east-central Texas are characterized as verdine facies clay, largely comprised of the mineral odinite. Odinite is an iron and magnesium 1∶1, 7Å clay. It is the main constituent of the clay fecal pellets and matrix of the central MGB. Few occurrences of odinite, other than modern odinite, are described in the literature. The MGB provides an informative example of odinite in the geologic record.

Odinite was established as the main constituent in clay pellets of the MGB through several analytical methods. X-ray diffraction found a 7.2Å clay mixture. Unit cell calculation indicated odinite-1M to be the most reliable interpretation. EMPA analysis of clay pellets, directed by QEMSCAN textural maps, identified the chemical composition. Mössbauer spectral analysis found the oxidation state at 3∶1 Fe^3+^/Fe^2+^ ratio diagnostic of odinite. Thus, essential chemical data was obtained to calculate the average structural formula for clay pellets of the MGB as follows: Fe^3+^_0.89_ Mg_0.45_ Al_0.67_ Fe^2+^_0.30_ Ti_0.01_ Mn_0.01_)_ Σ = 2.33_ (Si_1.77_ Al_0.23_) O_5.00_ (OH)_4.00_. Odin and Bailey [16], [24] first described odinite in 1988, and they calculated a similar mineral formula that was slightly more Mg rich and Fe, Al poor.

Documentation of odinite and verdine facies clays in the MGB is an important finding of this report. The environmental conditions associated with modern verdine facies clay occurrences include tropical latitudes, nearby runoff with iron influx, and water depth between 15 and 60 m (locally in 5 m depth). The characteristic sea-floor conditions are normal to nearly normal salinity, positive Eh, elevated sea-floor temperature and common association with fecal pellets. Circulating, winnowing currents are required to stir the sediment slightly and provide oxygenation. It is presumed that paleoenvironmental conditions were comparable during the Middle Eocene accumulation of authigenic verdine clays in the MGB.

The contrasting mineralogy between the central and the top of the MGB reflects a change in environmental conditions during its deposition. The central MGB has abundant pellets, comprised mostly of odinite, with lenses of shell concentrations and laminated sand. In contrast, the top of the MGB is a zone of concretions that are filled burrows comprised of pellets and detritus with siderite and apatite that formed as cementing agents. Glauconitic minerals are very sparse in both the central and top of the MGB and are probably allochthonous. A change in environmental conditions is reflected in a composite ichnofabric where deeper water biogenic activity in the central MGB is replaced by shallow water to intertidal burrowers in a firmground at the top of the MGB. The firmground was subsequently submerged and cemented.

The paleoenvironmental interpretation for the geologic section at Stone City Bluff has been the subject of numerous studies. This contribution of detailed mineralogy and geochemistry, although focused on the fecal pellets of the MGB, offers key information about processes during its deposition. With the identification of authigenic odinite or its alteration products, this study complements findings of previous studies. The MGB represents an ancient verdine facies paleoenvironment, which has implications for paleoecology, sedimentation rate, sequence stratigraphy and paleoclimate. It provides a coordinated approach to paleoenvironmental understanding of the dynamic depositional environment of the MGB at Stone City Bluff.

## Supporting Information

Figure S1 X-ray diffraction pattern of oriented 2 µ clay from bulk sample TX-8, (air dried, glycolated, heated to 375° and heated to 500°C).
(TIF)

Figure S2 X-ray diffraction pattern of oriented 2 µ, clay from bulk sample TX-9, (air dried, glycolated, heated to 375° and heated to 500°C).
(TIF)

Figure S3 X-ray diffraction patterns of randomly oriented powders TX-8, TX-18, and TX-9. Minerals of the verdine facies at the 060 diffraction peak are between1.56Å (59.23° 2θ) and 1.51Å (61.35° 2θ). Quartz at the 121- (*hkl*) peak is 1.54Å (59.970° 2θ).
(TIF)

Figure S4 Mössbauer Spectrum TX-9 & W, matrix & pellets.
(TIF)

Table S1 EMPA analyses for all odinite raw data in oxides and apfu.
(XLSX)

Table S2 EMPA analyses for all other clays, including glauconitic minerals in oxides.
(XLSX)
