# Supplementary figures and images for: Mineralogy and Geochemistry of the Main Glauconite Bed in the Middle Eocene of Texas: Paleoenvironmental Implications for the Verdine Facies
Source: PLoS One. 2014 Feb 4;9(2):e87656. doi: 10.1371/journal.pone.0087656 (PMC3913656; doi:10.1371/journal.pone.0087656)

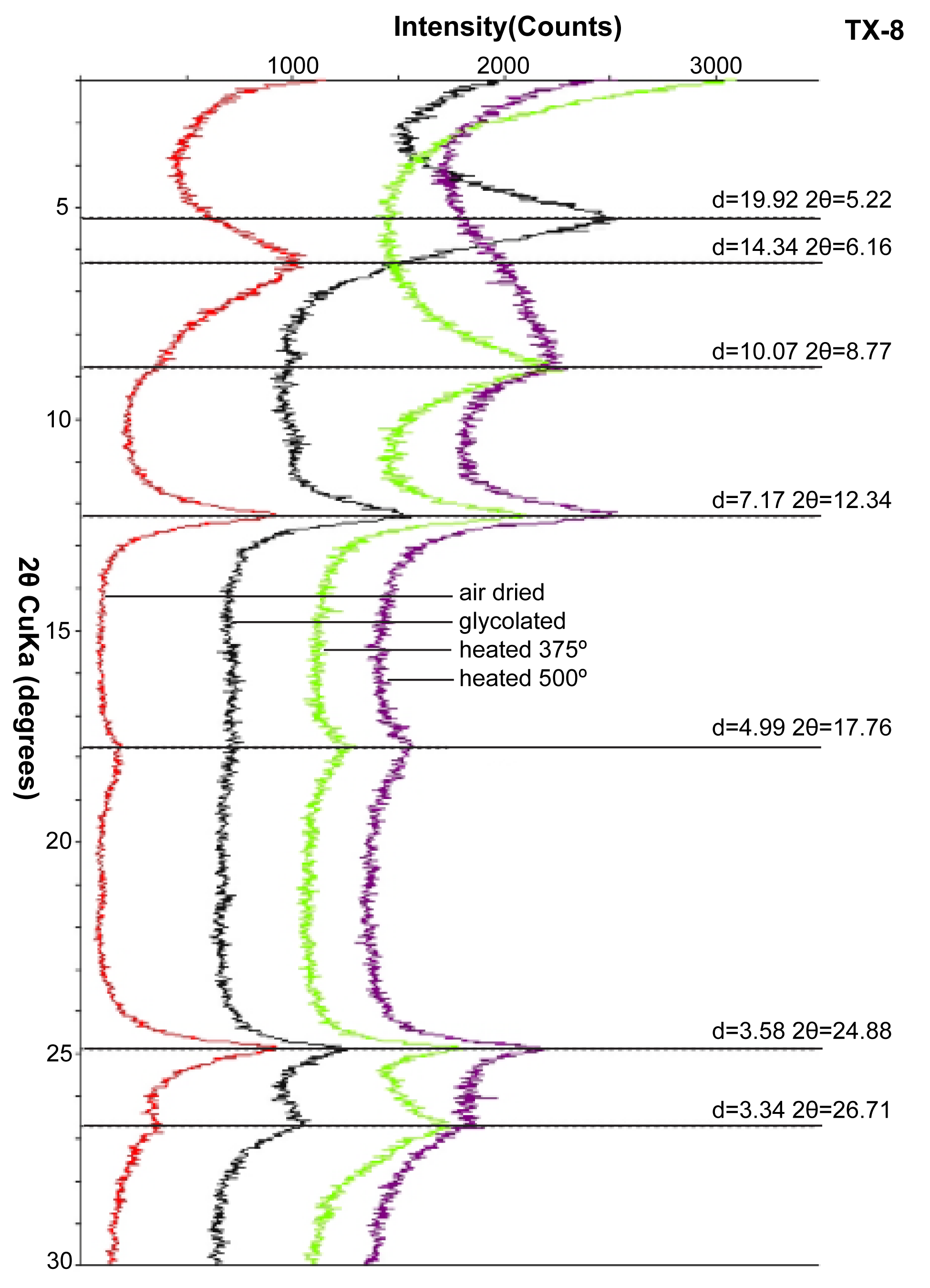

Supplement: Figure S1 — X-ray diffraction pattern of oriented 2 µ clay from bulk sample TX-8, (air dried, glycolated, heated to 375° and heated to 500°C). (TIF) [file pone.0087656.s001.tif]

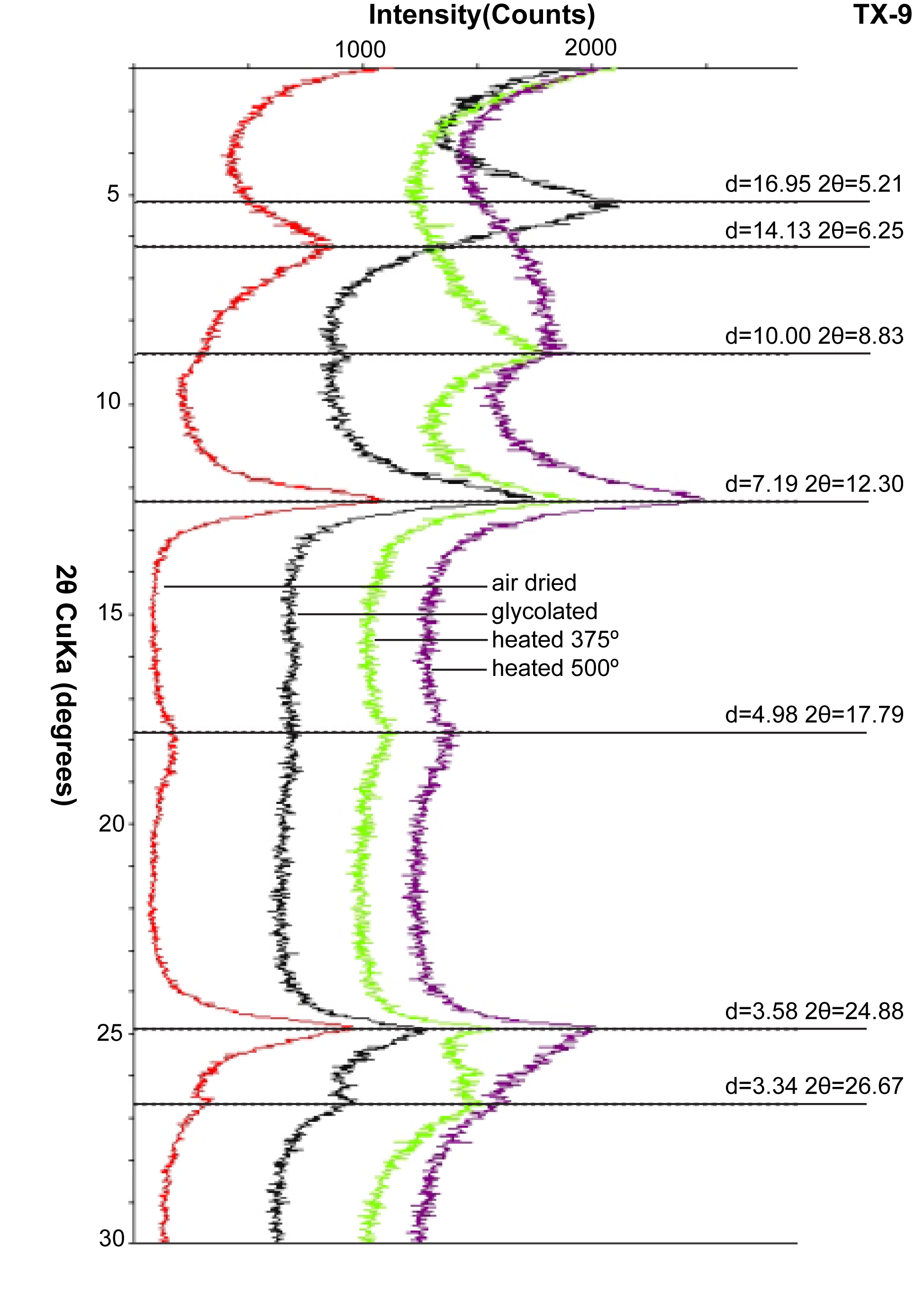

Supplement: Figure S2 — X-ray diffraction pattern of oriented 2 µ, clay from bulk sample TX-9, (air dried, glycolated, heated to 375° and heated to 500°C). (TIF) [file pone.0087656.s002.tif]

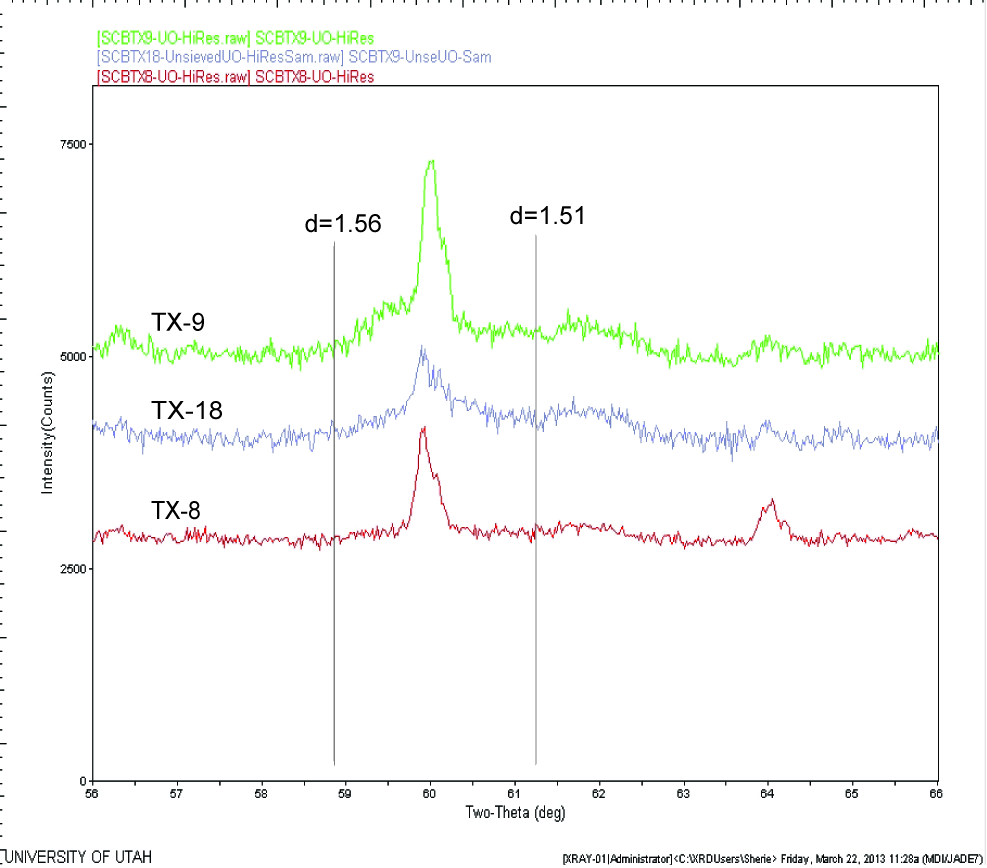

Supplement: Figure S3 — X-ray diffraction patterns of randomly oriented powders TX-8, TX-18, and TX-9. Minerals of the verdine facies at the 060 diffraction peak are between1.56Å (59.23° 2θ) and 1.51Å (61.35° 2θ). Quartz at the 121- (hkl) peak is 1.54Å (59.970° 2θ). (TIF) [file pone.0087656.s003.tif]

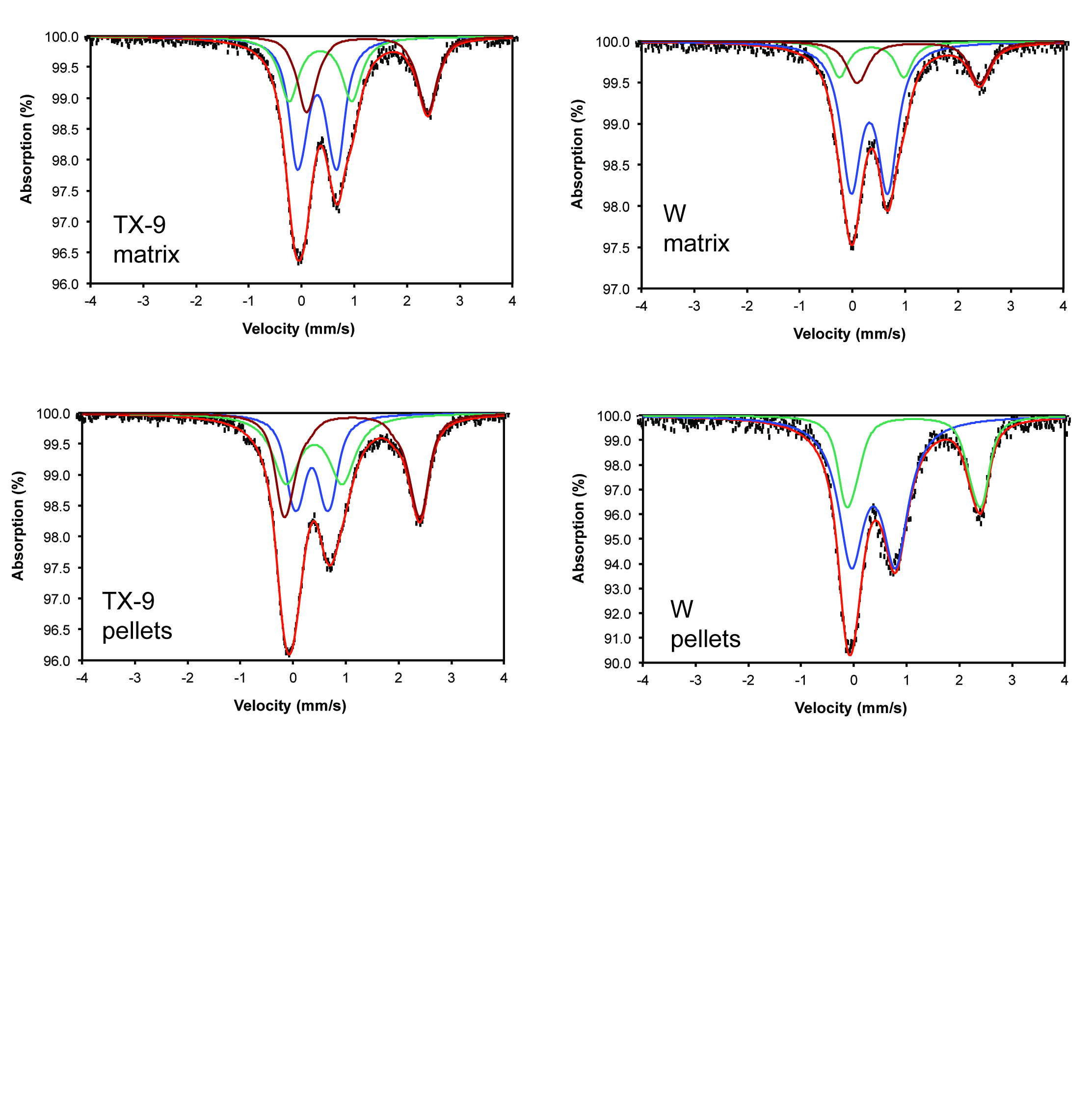

Supplement: Figure S4 — Mössbauer Spectrum TX-9 & W, matrix & pellets. (TIF) [file pone.0087656.s004.tif]
